# Morphological and molecular characterisation, and phylogenetic position of *X. browni* sp. n., *X. penevi* sp. n. and two known species of *Xiphinema americanum*-group (Nematoda, Longidoridae)

**DOI:** 10.3897/zookeys.574.8037

**Published:** 2016-03-28

**Authors:** Stela Lazarova, Vlada Peneva, Shesh Kumari

**Affiliations:** 1 Department of Animal Biodiversity and Resources, Institute of Biodiversity and Ecosystem Research, Bulgarian Academy of Sciences 2, Gagarin Street, 1113 Sofia, Bulgaria; 2 Division of Plant Health, Crop Research Institute, Drnovská 507, Ruzyně, 16106 Prague 6, Czech Republic

**Keywords:** Bayesian Inference, Bulgaria, Cytochrome c oxidase subunit 1, Czech Republic, Morocco, Nicotinamide dehydrogenase subunit 4, phylogeny, ribosomal DNA, Slovakia

## Abstract

Using ribosomal (18S, ITS1, ITS2, D2-D3 expansion segments of 28S rDNA) and mitochondrial (partial *cox*1 and *nad*4) DNA markers in a study of several populations of *Xiphinema americanum*-group from Europe and Morocco, two cryptic species *Xiphinema browni* **sp. n.** (formerly reported as *Xiphinema pachtaicum*) and *Xiphinema penevi* **sp. n.** were revealed. The species are described, illustrated and their phylogenetic relationships discussed. The first species is most similar to *Xiphinema parasimile* and is a member of *Xiphinema simile* species complex. The phylogenetic reconstructions inferred from three molecular markers (18S, D2-D3 28S rDNA and *cox*1) showed that *Xiphinema penevi* **sp. n.** is part of *Xiphinema pachtaicum*-subgroup and is closely related to *Xiphinema incertum*, *Xiphinema pachtaicum*, *Xiphinema parapachydermum*, *Xiphinema plesiopachtaicum*, *Xiphinema astaregiense* and *Xiphinema pachydermum*. Also, a separate “*Xiphinema simile*-subgroup”, outside the *Xiphinema pachtaicum*-subgroup and so far consisting only of the parthenogenetic species *Xiphinema simile*, *Xiphinema parasimile*, *Xiphinema browni* **sp. n.** and probably *Xiphinema vallense* was formed. New primers for amplification and sequencing of part of the *nad*4 mitochondrial gene were designed and used.

## Introduction

The *Xiphinema americanum*-group is a well defined natural complex of species (Lamberti et al. 2000, Coomans et al. 2001, He et al. 2005b) with high significance to agriculture caused by the ability of several species to transmit economically important plant viruses (McFarlane et al. 2002), although there are controversial opinions defining the group (Archidona-Yuste et al. 2016). Even for experienced nematologists species delimitation within this group is challenging because they have rather similar morphology and metrics, and the existing keys (Lamberti et al. 2000, 2004) do not always allow species differentiation and identification. During the last decade wide usage of DNA sequencing in *Xiphinema* taxonomy including this group revealed the existence of a number of cryptic species (Gutiérrez-Gutiérrez et al. 2010, 2012, Archidona-Yuste et al. 2016). This was the case with several populations from the Czech Republic and Slovakia (Kumari et al. 2005, 2010b) originally identified as *Xiphinema pachtaicum* (Tulaganov, 1938) and one population from Morocco provisionally also determined as *Xiphinema pachtaicum*. The objectives of the present study were: i) to characterise populations from the Czech Republic, Slovakia and Morocco both morphologically and genetically; ii) to sequence populations of *Xiphinema pachtaicum* and *Xiphinema parasimile* Barsi & Lamberti, 2004 from Bulgaria for comparison; iii) to clarify phylogenetic relationships of identified species using ribosomal and mithochondrial DNA.

## Material and methods

### Sampling, nematode isolation and processing

The *Xiphinema* specimens examined originated from various localities in the Czech Republic (Kurdějov, Mohyla míru and Sokolnice, grapevines), Slovakia (Moča, grapevine), Bulgaria (Balgarene village, pear tree, Vinogradets vicinity, vineyard) and Morocco (Ifrane, holm oak tree). Details of the soil sampling, nematode isolation and processing for Czech and Slovakian populations are given in Kumari et al. (2005, 2010b). A decanting and sieving technique was used for extracting nematodes from soil samples from Bulgaria and Morocco. *Xiphinema* specimens recovered were heat killed at 55°C for two minutes, fixed in a 4% formalin, 1% glycerol solution, processed to anhydrous glycerol (Seinhorst 1959), and mounted on glass microscope slides. Drawings were prepared using an Olympus BX51 compound microscope with differential interference contrast (DIC). Photographs were taken using an Axio Imager.M2-Carl Zeiss compound microscope with a digital camera (ProgRes C7) and specialised software (CapturePro Software 2.8). Measurements were made using an Olympus BX41 light microscope, a digitising tablet (CalComp Drawing Board III, GTCO CalCom Peripherals, Scottsdale, AZ, USA), and computer Digitrak 1.0f programme (Philip Smith, Scottish Crop Research Institute, Dundee, UK).

### DNA extraction, amplification and sequencing

Individual nematodes from Bulgaria, Morocco (DESS-preserved), Czech Republic and Slovakia (1M NaCl-preserved) were mounted on temporary slides containing glass beads and after taking measurements and photomicrographs the slides were dismantled, individual nematodes removed, and added in 0.25 M NaOH to digest overnight and thereafter heated to 99°C for 3 min. Afterwards 10 μl of 0.25 M HCl, and 5 μl each of 0.5 M Tris-HCl (pH 8) and 2% Triton X-100 were added and the mixture was incubated for another 3 min at 99°C (Stanton et al. 1998). Finally, the DNA suspension was cooled and the DNA was either used directly for PCR or stored at -20°C until template was needed for PCR reactions. Genomic DNA which was prepared by Kumari et al. (2010b) was also used in this study.

Six regions (18S, ITS1, ITS2, D2-D3 expansion segments of 28S, *cox*1 and *nad*4) of ribosomal and mitochondrial DNA were amplified and sequenced. Primer sequences and references to the primers are given in Table 1. The 18S gene of the Czech population was amplified by using primers SSU_F_04+SSU_R_09 (first fragment), SSU_F_22+SSU_R_13 (second fragment) and SSU_F_23+SSU_R_81 (third fragment). The 18S gene of other populations was amplified by using primer combination 988F+1912R (first fragment) and 1813F+2646R (second fragment).

**Table 1.** Primers used to amplify ribosomal and mitochondrial DNA.

Gene	Primer name	Direction	Primer sequence 5′ - 3′	Reference
18S	SSU_F_04	forward	GCT TGT CTC AAA GAT TAA GCC	Blaxter et al. (1998)
18S	SSU_R_09	reverse	AGC TGG AAT TAC CGC GGC TG	Blaxter et al. (1998)
18S	SSU_F_22	forward	TCC AAG GAA GGC AGC AGG C	Blaxter et al. (1998)
18S	SSU_R_13	reverse	GGG CAT CAC AGA CCT GTT A	Blaxter et al. (1998)
18S	SSU_F_23	forward	ATT CCG ATA ACG AGC GAG A	Blaxter et al. (1998)
18S	SSU_R_81	reverse	TGA TCC WKC YGC AGG TTC AC	Blaxter et al. (1998)
18S	988F	forward	CTC AAA GAT TAA GCC ATG C	Holterman et al. (2006)
18S	1912R	reverse	TTT ACG GTC AGA ACT AGG G	Holterman et al. (2006)
ITS1	pXb101	forward	TTG ATT ACG TCC CTG CCC TTT	Vrain et al. (1992)
ITS1	ChR	reverse	ACG AGC CGA GTG ATC CAC CG	Cherry et al. (1997)
ITS2	WDF	forward	AGA CAC AAA GAG CAT CGA CT	Kumari et al. (2009)
ITS2	pXb481	reverse	TTT CAC TCG CCG TTA CTA AGG	Vrain et al. (1992)
D2-D3	D2A	forward	ACA AGT ACC GTG AGG GAA AGT TG	Nunn (1992)
D2-D3	D3B	reverse	TCG GAA GGA ACC AGC TAC TA	Nunn (1992)
*cox*1	COIF	forward	GAT TTT TTG GKC ATC CWG ARG	He et al. (2005a)
*cox*1	XIPHR2	reverse	GTA CAT AAT GAA AAT GTG CCA	Lazarova et al. (2006)
*nad*4	CDF	forward	AAA AAG ATG GTA TTG GAG	Kumari and Cesare (2013)
*nad*4	CDR	reverse	GCA CAT GTA GAA GCT AGT	Kumari and Cesare (2013)
*nad*4	nadpachF	forward	ATA GAA GCA TTA CCA ACT A	This study
*nad*4	nadpachR	reverse	TAG TAC CAG AGG ATC AAT A	This study

Initially partial *nad*4 gene was amplified with the primers CDF+CDR but only one specimen was amplified using these primers. A pair of new primers (nadpachF+nadpachR) was designed using online software PRIMER 3 (http://frodo.wi.mit.edu/) from the sequences which were amplified by (CDF+CDR). For final analysis all specimens and populations of *Xiphinema browni* sp. n. from the Czech Republic and Slovakia were amplified and sequenced by using nadpachF + nadpachR primers.

The PCR reaction was performed in 25 μl total volume containing 1 PCR bead (GE Healthcare, Buckinghamshire, UK), 20.5 μl double distilled sterile water, 2.0 μl of each primer (10pmol/μl) (synthesized by Generi Biotech, Hradec Králové, Czech Republic), and 0.5 μl of DNA added as a template for PCR. A negative control (sterilized water) was included in all PCR experiments. The cycling profile for all ribosomal DNA and mtDNA markers was as described by Kumari and Subbotin (2012) and by He et al. (2005a), respectively. All PCR reactions were performed in a DNA Engine PTC–1148 thermal cycler (Bio-Rad). Aliquots of PCR were analysed by gel electrophoresis and the remaining products were purified using High Pure Product Purification kit (Roche Diagnostics GmbH, Mannheim, Germany) and sequenced in both directions using each primer pair one forward and one reverse (Macrogen, Netherlands). Sequencher^TM^ 4.8 (Genes codes. Corp., Ann Arbor, MI, USA) was used to assemble and view each sequence and check for base-calling errors. Accession numbers of all sequences are given in Table 2.

**Table 2.** NCBI accession numbers of representative individual specimens for ribosomal and mitochondrial DNA.

Species	*Xiphinema browni* sp. n				*Xiphinema pachtaicum*	*Xiphinema parasimile*	*Xiphinema penevi* sp. n.
Country	Czech Republic			Slovakia	Bulgaria	Bulgaria	Morocco
Locality	Kurdějov	Mohyla míru	Sokolnice	Moča	Balgarene	Vinogradets	Ifrane
Isolate	NSB1	NSB2	NSB3	NSB4	NSB5	NSB6	NSB7
18S	KU250135	KU250136	KU250137	KU250138	KU250139	KU250140	KU250141
18S+ITS1	KU250142	KU250143	KU250144	**NA**	**NA**	**NA**	**NA**
5.8S+ITS2+28S	KU250145	KU250146	KU250147	KU250148	KU250149	**NA**	KU250150
D2/D3	KU250151	KU250152	KU250153	KU250154	KU250155	KU250156	KU250157
*cox1*	GU222424*	*	*	KU250158	**NA**	KU250159	**NA**
*nad4*	KU250160	KU250161	KU250162	KU250163	**NA**	**NA**	**NA**

* Kumari et al. (2010); NA = not acquired

### Sequence and phylogenetic analyses

A BLAST (Basic Local Alignment Search Tool) search at NCBI (National Center for Biotechnology Information) was performed using the obtained sequences as queries to confirm their nematode origin and to identify the most closely related nematode sequences. Sequences revealing high similarity to those obtained here were included in the phylogenetic analyses of both ribosomal and mitochondrial gene regions (Neilson et al. 2004, Oliveira et al. 2004, He et al. 2005b, Gozel et al. 2006, Holterman et al. 2006; Lazarova et al. 2006, Kumari et al. 2009, Gutiérrez-Gutiérrez et al. 2010, Kumari et al. 2010a, Kumari et al. 2010b, De Luca and Agostinelli 2011, Gutiérrez-Gutiérrez et al. 2011a, Gutiérrez-Gutiérrez et al. 2011b, Meza et al. 2011, Sakai et al. 2011, Gutiérrez-Gutiérrez et al. 2012, Kumari and Subbotin 2012, Sakai et al. 2012, Kumari and Cesare 2013, Tzortzakakis et al. 2014, Getaneh et al. 2015, etc). Sequence numbers are presented in the trees. The multiple sequence alignments (MSA) of all datasets were performed using the GUIDANCE2 Server available at http://guidance.tau.ac.il/ (Sela et al. 2015). All three alignment algorithms (MAFFT, PRANK and ClustalW) were tested and the MSAs having highest alignment confidence scores were used for ITS phylogenetic reconstructions. Subsequently, the MSAs were manually optimised and trimmed using MEGA 6 (Tamura et al. 2013). The phylogenetic reconstructions were performed using the Bayesian Inference (BI) algorithm implemented in MrBayes 3.2.5. (Huelsenbeck and Ronquist 2001; Ronquist et al. 2012) using the General Time Reversible model plus Gamma distribution rates (GTR + G). The Bayesian MCMC tree searches were run using default heating parameters for 2 000 000 generations with a sample frequency of 1000 generations. The first 25% of the chains discarded as burning and the remaining 75% trees kept to summarise the tree topology, branch lengths, and posterior probabilities (PP) of branch support. Convergence diagnostic values were calculated every 1000 generations with a predefined stop value equal to 0.01. A single strict consensus tree was visualised using FigTree v1.4.2 graphical viewer. Posterior probabilities values of ≥0.80 were considered as credible support values for nodes.

## Taxonomy

### Xiphinema browni sp. n.

Taxon classificationAnimaliaDorylaimidaLongidoridae

http://zoobank.org/E385F7F7-2C78-4D54-BC57-0D24EDD43CB8

Figures 1 , 2 , 3 , 4 , 5 , 6 , 7 , 8 , 15 , 16 , 17 , 18

Xiphinema pachtaicum (Tulaganov, 1938) Kirjanova, 1951 apud Kumari et al. 2005, syn. n.

#### Measurements.

See Tables 3–5.

**Table 3.** Morphometrics of *Xiphinema browni* sp. n (localities in the Czech Republic and Slovakia) and *Xiphinema pachtaicum* (Bulgaria). All measurements in micrometres, except ratios given as mean ± standard deviation (range).

		*Xiphinema browni* sp. n					*Xiphinema pachtaicum*
Locality		Kurdějov	Sokolnice		Mohyla míru	Moča	Balgarene
Plant host		grapevine	grapevine		apple	grapevine	pear
n	Holotype	50 females	20 females	male	12 females	4 females	6 females
L	1904	2031±123 (1798–2408)	1886±89	1849	1972±90	1715±142	1735±232
			(1751–2099)		(1785–2079)	(1603–1922)	(1522–2015)
a	57.8	69.3±5.16	60.5±4.4	73.9	60.1±3.14	69.5±6.57	58.7±4.9
		(56.9–81.3)	(52.3–69.9)		(55.6–64.5)	(63.6–76.3)	(53.3–65.7)
b	6.7	7.3±0.76	6.9±0.38	6.9	7.0±0.32	8.2, 6.8	5.9±0.5
		(6.1–8.7)	(6.4–7.9)		(6.4–7.4)		(5.3–6.4)
c	64.8	69.9±6.22	65.8±5.71	54.4	64.9±3.41	61.6±8.72	58.2±8.3
		(54.7–83.0)	(56–79.6)		(58.5–70.3)	(53.4–73.9)	(50.9–66.3)
c’	1.9	1.78±0.12	1.82±0.14	1.89	1.8±0.08	1.8±0.17	1.7±0.1
		(1.53–2.07)	(1.61–2.13)		(1.6–1.9)	(1.5–1.9)	(1.6–1.8)
V/Spicule length	56.1	55±1.30	55±1.71	29.0	55.4±1.15	55.5±1.16	58.6±1.4
		(52.3–58.5)	(49–57)		(53.8–58.1)	(53.8–56.4)	(57.0–60.4)
Odontostyle	84	83±2.2	79±2.6	76	82±3.39	77±4.69	84.2±3.7
		(78–86)	(74–83)		(73–85)	(72–81)	(78–88.5)
Odontophore	43	42±1.69	41±0.91	38	43±1.88	38±3.30	48.9±2.1
		(38–48)	(39–43)		(39–46)	(35–42)	(46–51)
Oral aperture to	72	71±2.56	68±2.35	67	71±1.68	66±5.06	76.8±3.4
guide ring		(65–75)	(63–72)		(67–73)	(60–72)	(73–80)
Tail length	29	29±1.94	29±2.24	34	30±0.82	28±1.63	29.8±0.9
		(25–33)	(24–32)		(29–32)	(26–30)	(28–30)
Length of hyaline	8	8±1.28	8±1.22	10	8±0.68	8±1.41	8.7±1.0
part		(6–12)	(6–10)		(7–9)	(7–10)	(8–10)
Body diam. at:	8	8±0.58	8±0.51	9	9±0.43	8±0.50	8.8±0.2
- lip region		(8–10)	(8–9)		(8.5–10)	(7–8)	(8.5–9)
- guiding ring	22	20±0.67	19±0.49	19	22±1.44	19±1.41	21.5±1.0
		(19–21)	(19–20)		(19.5–24)	(18–21)	(20.5–23)
- base of pharynx	29	26±1.58	26±2.41	23	28±1.69	23.1, 23.8	26.5±1.2
		(22–32)	(19–20)		(25–30)		(25–28)
- mid body	33	29±2.78	31±2.58	25	34±2.66	25±1.89	28.9±2.1
		(25–38)	(26–37)		(29–38.5)	(22–26)	(26–32)
- anus	16	16±0.97	16±0.92	18	17±0.89	16±0.96	17.1±1.0
		(14–19)	(15–18)		(16–19)	(15–17)	(16–19)
- beginning of	7.5	7±1.11	7±0.62	8		8±1.50	8.7±0.0
hyaline part		(5–10)	(6–8)			(6–9)	(9–9)

**Table 4.** Pharyngeal characters of females of *Xiphinema americanum* group species studied from different localities.

	***Xiphinema browni* sp. n.**			***Xiphinema pachtaicum***	***Xiphinema penevi* sp. n.**	***Xiphinema parasimile*Lazarova et al. (2008)**	
Locality Character	Kurdějov, Czech Republic	Sokolnice, Czech Republic	Mohyla míru, Czech Republic	Balgarene, Bulgaria	Ifrane, Morocco	Vinogradets, Bulgaria	Ralja, Serbia Paratypes
n	50	20	5	5	6	14	5
Pharynx length (μm)	278.0±17.9 (236–309)	272.0±12.1 (247–297)	271.4±20.1 (234–294)	303.5±13.5 (291–317)	274.4±29.2 (229–308)	250.5±21.0 (233–311)	282.2±2.1 (260–310)
Bulbus length (μm)	60±3.48 (53–69)	59±3.02 (56–67)	60±2.88 (56–63)	75, 77, 80	68.4±2.7 (65–72)	59.8±3.5 (55.5–68)	61.6±4.8 (56–63)
Bulbus width (μm)	13±1.30 (9–16)	13±1.21 (11–16)	14±1.14 (12–15)	15, 15, 16	13.6±0.8 (12–14)	12.0±0.6 (11–13)	14.4±0.5 (14–15)
Bulbus length/ Pharynx length (%)	21.7± 1.7 (17.4–27.1)	21.8±1.6 (19.5–27.1)	22.3±1.3 (20–24)	25.6±07 (25–26)		24.2±2.3 (19–28) n=11	21.8±0.7 (20.7–22.6)
DN* (%)	17.5±1.9 (15.3–21.1) n=6	13.1±2.3 (12.7–17.3) n=5	12.5, 13.0, 14.9	9.3, 10.3	11.4±1.4 (9.9–12.9)	16.7±3.3 (13.6–18.6) n=8	16.5–17.7
DO* (%)	10.9±1.7 (8.8–13.8) n=6	7.9±3.8 (5.5–15.6) n=5	11.9±1.8 (8.8–13.3) n=5	11.5, 12.0	12.1±1.6 (9.9–13.2)	11.1, 13.6	11.6–14.6
SVN1* (%)		53.9±1.6 (51.8–55.0)	55.6, 54.4	60.3	56.7±2.0 (53.8–58.8)		55.3–59.7
SVN2* (%)		53.2			58.7±2.9 (55.4–61.0)		57.3–60.1
SVO (%)	74.2±1.9 (71.4–75.4) n=4	74.9±3.3 (67.6–76.4) n=5	68.5, 71.1, 71.8	72.0, 74.4	75.4±2.4 (73.5–79.4)		
Glandularium ** (μm)	48.5±1.9 (46–52) n= 8	50.6±2.3 (48–51) n=5	53, 48, 46	68, 70, 70	61.9±3.1 (57–65) n=8	49.9±1.4 (48–52) n=8	52.3±2.2 (52–56)

Terminology adopted by Loof and Coomans (1972)*; and Andrássy (1998)**.

**Table 5.** Measurements of uteri (including ovejector), ovejector and vaginal parts. All measurements in micrometres presented as mean ± standard deviation (range).

	Characters Locality	Anterior uterus	Posterior uterus	Ovejector	Vagina length	*Pars distalis vaginae*	*Pars proximalis vaginae*
***Xiphinema browni***	Kurdějov	42.1±5.7 (35–54) n=8	38.9±5.0 (31–43) n=7	26	12.9±1.4 (11–15) n=9	6.7±1.1 (5.5–8.5) n=6	13.7±0.6 (13–14) n=3
	Sokolnice	45.5±3.7 (38–46) n=4	46.0±4.0 (40–49) n=4	30.5		5, 6, 6	8.5, 10, 10
	Mohyla míru	39, 40, 50	39, 41.5, 44	26, 33	12.5±1.0 (11–14) n=5	5, 6	10, 10
***Xiphinema penevi***	Ifrane	52.2±9.0 (36–68)	52.3±4.3 (46–58)	26		8.9±0.3 (8–9)	10.6±1.2 (8–13)
***Xiphinema pachtaicum***	Balgarene	40, 48	42, 49, 50	37	13, 14, 15	9, 9	12, 12
***Xiphinema parasimile***	Vinogradets	33.1±0.4 (30–38) n=13	31.2±0.7 (24–39) n=13	29.4±4.3 (26–33.5) n=10	14.5±1.7 (13–15) n=17	7.4±0.5 (7–8) n=15	7.4±0.5 (7–9) n=19
	Ralja,Trešna paratypes	40.0±11.3 (27–46) n=3	-	-	14.5±1.05 (13–16) n=5	7.8±0.8 (7–8.5) n=3	8.75±0.3 (8.5–9) n=4
***Xiphinema simile***	Srebarna, Bulgaria	18.8±2.8 (14–21) n=6	18.5±2.4 (15–20) n=6	36.3±6.4 (29–41) n=3	14.8±1.3 (13–16) n=5	5.8±0.4 (5.5–6) n=8	9.5±0.9 (8.5–11) n=7
	Kalimok- Brashlen Bulgaria	21.8±1.9 (16.5–24) n=14	21.5±1.8 (19–24) n=14	43.1±3.1 (36.5–48) n=12	16.8±0.8 (15–18) n=15	6.4±0.65 (5.5–7) n=17	8.6±0.5 (8–10) n=17
	Orlyane Bulgaria	21.75±2.2 (17–24) n=7	22.1±2.3 (19–26) n=7	43.8±4.2 (36–50) n=7	16.9±1.1 (15–18) n=8	6.05±0.6 (5.5–7) n=11	9.05±0.8 (8–10) n=10
	Kamen bryag Bulgaria	23.0±4.8 (18–30) n=5	24.2±4.15 (19–30) n=5	47.2±8.9 (37–60) n=5	15.9±1.8 (13–17) n=5	6.4±0.6 (6–7) n=5	9.8±0.8 (9–10) n=5

Data for *Xiphinema parasimile* and *Xiphinema simile*, Lazarova et al. (2008).

#### Description.

*Females*. Body slender C to open spiral shaped. Cuticle with fine transverse striae. Thickness of the cuticle at postlabial region 1–1.5 μm, 1.5 rarely 2 μm at mid-body and 2 μm at post-anal region. Labial region set-off from the rest of the body by a constriction, expanded, rounded laterally, 5.0±1.1 (4–7) μm high. Amphideal fovea hardly visible, funnel-shaped, its opening *c*. 5 μm (50%) wide visible posterior the constriction level. Distance between first and second guide ring in specimens with retracted odonostyle 5–10 μm long. Odontophore with moderately developed basal flanges 6.1±0.6 (5.5–7) μm wide. A small vestigium observed occasionally in slender part of pharynx. Pharyngeal characters presented at Table 4. Dorsal pharyngeal gland nucleus 2 μm diam. Ventrosublateral nuclei barely visible. Rectum 20.8 ± 1.5 (18–23) μm, n=7, or *c* 1.3 times anal body diameter. Reproductive system amphidelphic, symbiont bacteria present in the ovaries. Separate uteri and ovejector present (Table 5), oviduct 90.5±13.0 (68–101) μm; vagina bell-shaped 39.5% of the corresponding body width (33–50%, n=14), vulva post-equatorial. Numerous sperm observed in one female from Kurdějov (Figs 2B, 4B). Tail conical, dorsally convex, ventrally straight or slightly concave with narrowly rounded to pointed terminus. Two pairs of caudal pores.

**Figure 1.**
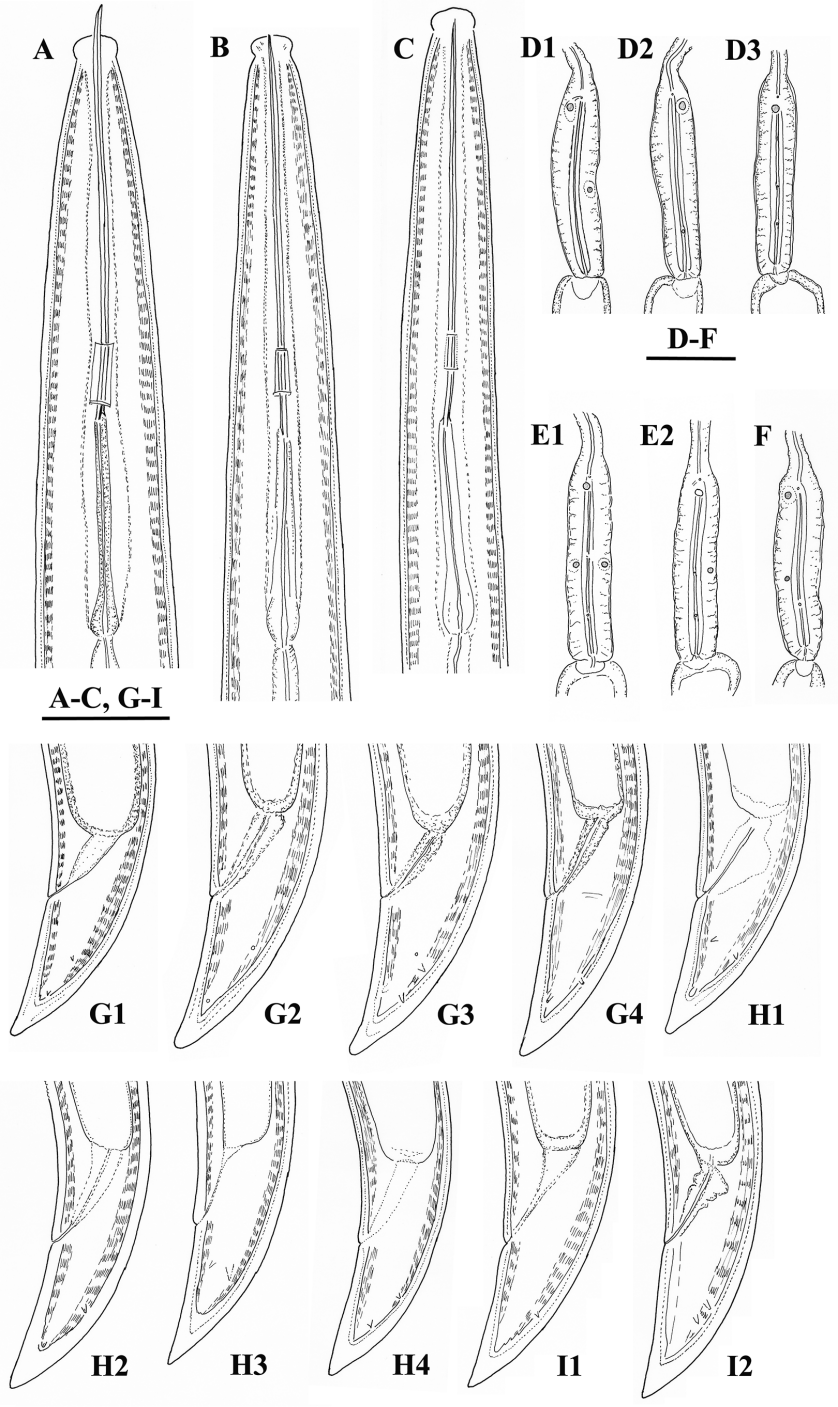
*Xiphinema browni* sp. n. Female: Variations in: **A–C** Anterior end **D–F** Pharyngeal bulbus **G–I** Tail shape **A, D, G** Kurdĕjov (type population) **B, F, I** Mohyla míru **C, E, H** Sokolnice. Scale bars: 25 μm

**Figure 2.**
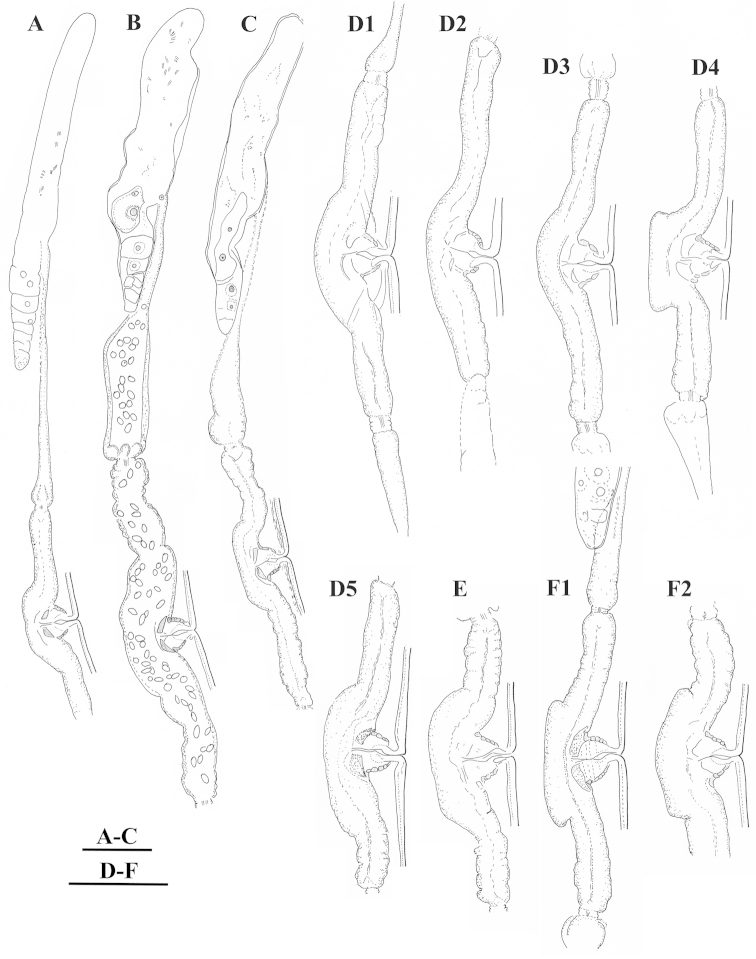
*Xiphinema browni* sp. n. Female: Variations in genital system: **A, B** Anterior genital branch **C** Posterior genital branch **D–F** Region of vagina and uteri **A, B, D** Kurdĕjov (type population) **C, F** Mohyla míru **E** Sokolnice. Scale bars: 25 μm.

**Figure 3.**
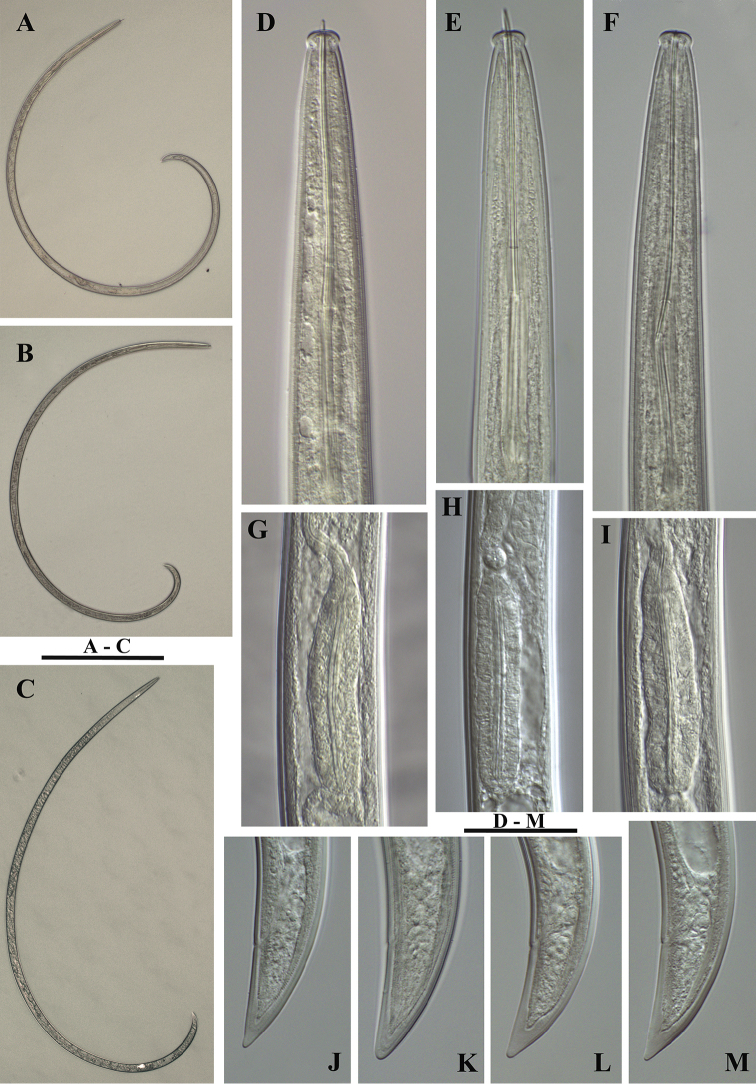
*Xiphinema browni* sp. n. **A–C** Entire body (**A, C** females **B** male); Female: **D–F** Anterior ends **G–I** Pharyngeal bulbus **J–M** Tail shape variation **A, F, I, M** Mohyla míru **B, E, H, L** Sokolnice **C, D, G, J, K** Kurdĕjov (type population). Scale bars: (**A–C)** 400 μm; (**D–M**) 30 μm.

**Figure 4.**
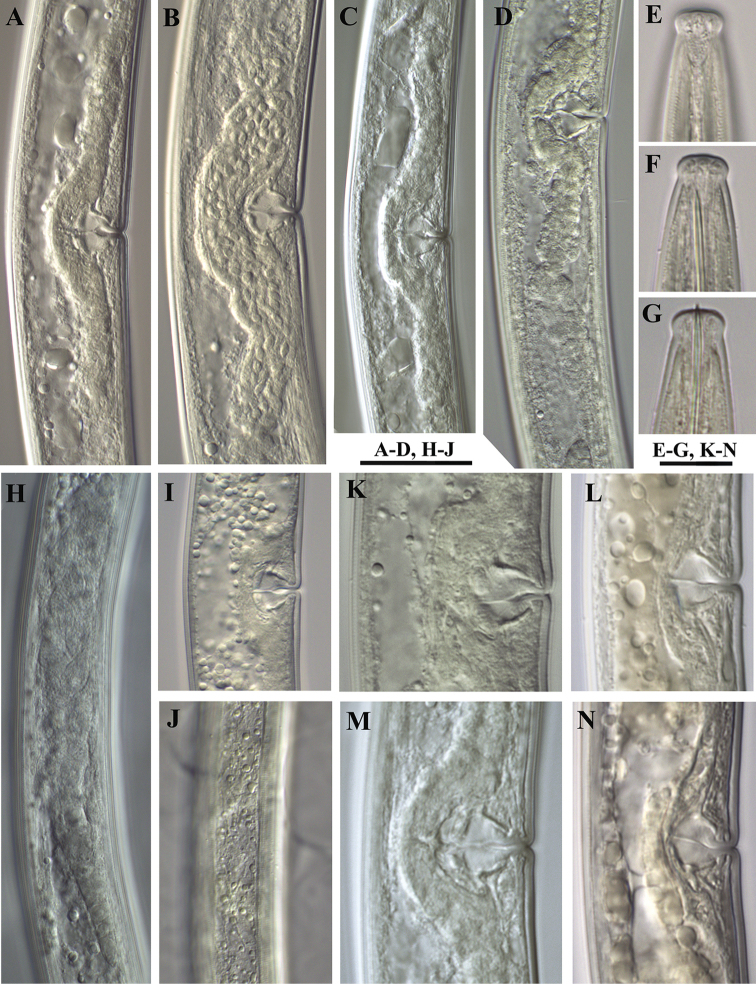
*Xiphinema browni* sp. n. Female: **A–D** Genital system (**B** uterus full with sperm) **E–G** Labial region (**E** Amphid **F** Female **G** Male) **H** Ovary with endosymbionts **I, K–N** Variations in vagina **J** Lateral field **A, B, H, I, J** Kurdĕjov **C, G** Sokolnice **D, K, M** Mohyla míru **E, F, L, N** Moča. Scale bars: 30 μm **(A–D, H–J)**; 12 μm **(E–G, K–N)**.

*Male*. Very rare. One specimen found in Sokolnice population. Male similar to the female with posterior region more strongly curved. Lip region and tail shape as in females, differences were observed within body width and tail length, which reflected **a** and **c**’ values. Spicules robust, slightly curved, lateral guiding piece 7 μm long. Adanal pair preceeded by a row of 5 irregularly spaced supplements, the two anteriormost weakly developed. Tail conoid, ventrally straight, dorsally convex with pointed terminus, caudal pores not visible. The slide of the only male specimen, described by Kumari et al. (2005), was subsequently damaged.

*Juveniles*. The scatter diagram based on functional and replacement odontostyle, and body length revealed the presence of four juvenile stages (Fig. 8). Tail shape and length similar in all stages and females with **c**’ slightly decreasing in successive stages (Kumari 2005, Fig. 3, Table 3).

**Figure 5.**
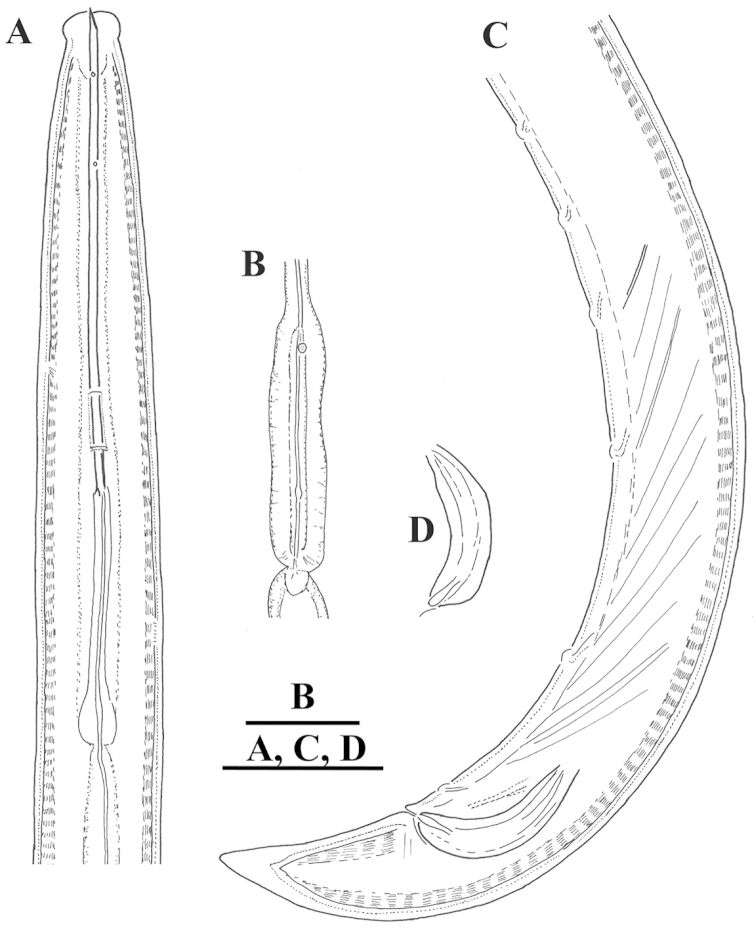
*Xiphinema browni* sp. n., Sokolnice. Male: **A** Anterior end **B** Pharyngeal bulbus **C** Posterior end **D** Spicules. Scale bars: 25 μm

**Figure 6.**
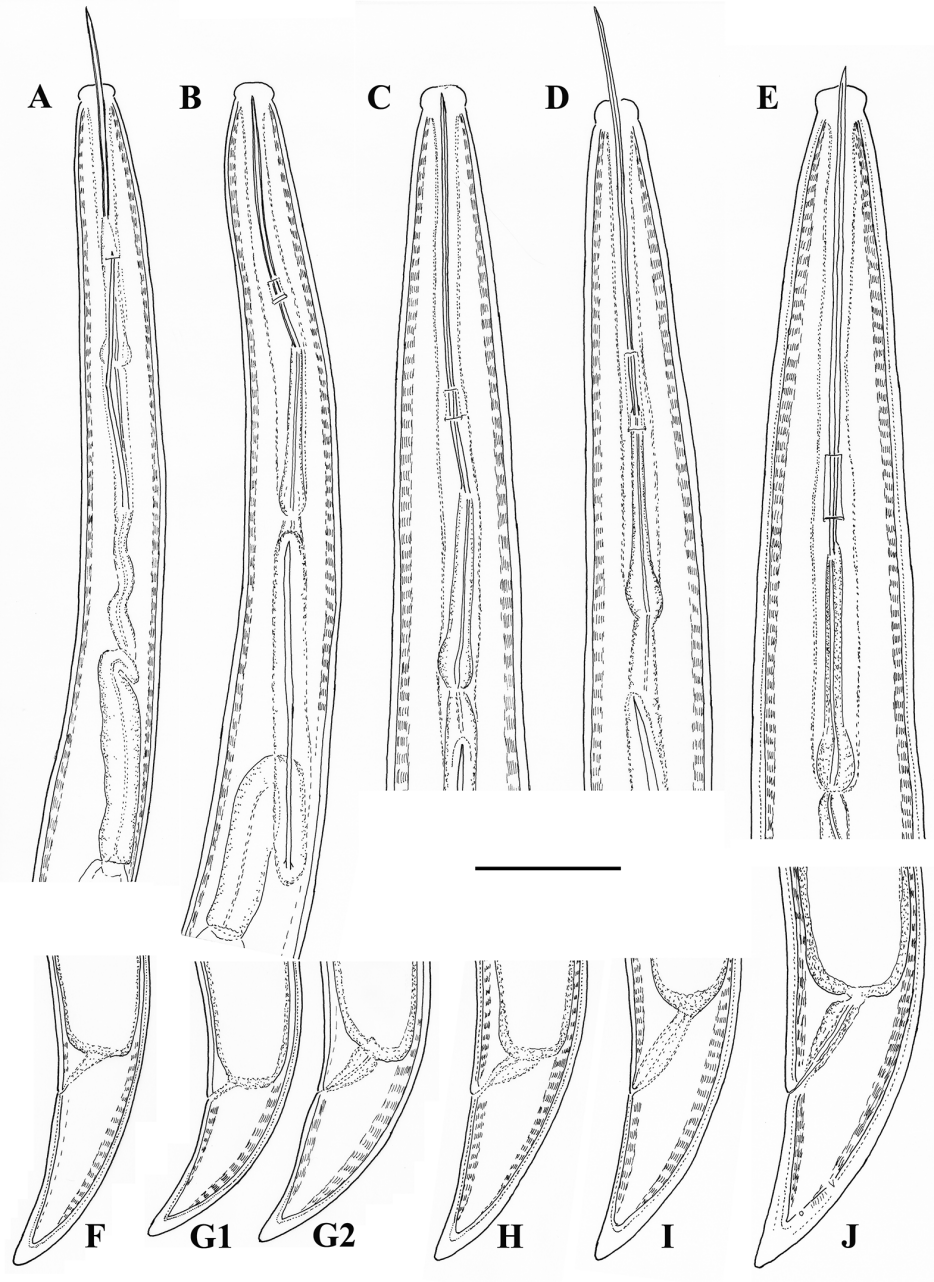
*Xiphinema browni* sp. n., Kurdĕjov. Juveniles and female: **A–E** Anterior ends of first- to fourth-stage juveniles and female **F–J** Tails of first- to fourth- juvenile stages and female. Scale bar: 25 μm

**Figure 7.**
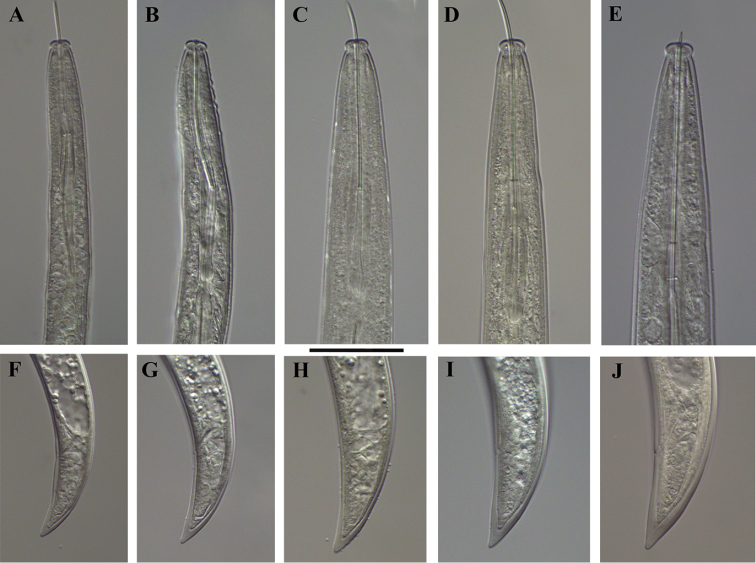
*Xiphinema browni* sp. n. Kurdĕjov. Juveniles and female: **A–E** Anterior ends of first- to fourth-stage juveniles and female **F–J** Tails of first to fourth juvenile stages and female (G1 and G2 – second-stage juvenile). Scale bar: 30μm.

**Figure 8.**
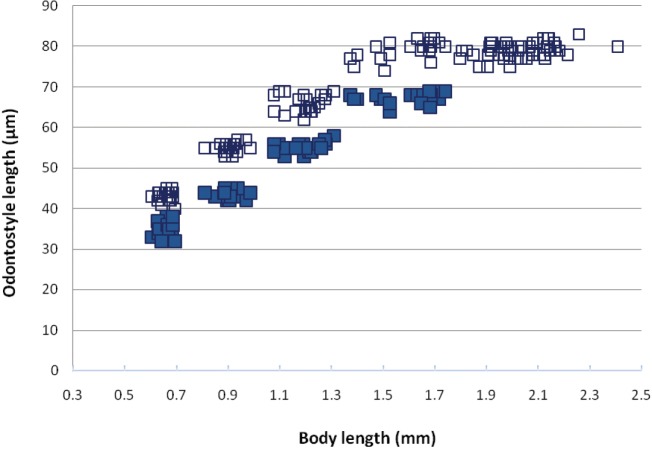
Scatter plot of odontostyle (■) and replacement odontostyle (□) against body length of *Xiphinema browni* sp. n. juveniles and females from Kurdĕjov population.

#### Type locality and plant association.

Kurdějov, Břeclav County, South Moravia, Czech Republic, associated with grapevine. Other localities: Mohyla míru, Brno-Venkov County, South Moravia, the Czech Repbulic, in the rhizosphere of apple trees; Sokolnice, Brno-Venkov County, South Moravia, the Czech Repbulic, in the rhizosphere of grapevine; Moča, Komárno County, Nitra, Slovak Republic, in the rhizosphere of grapevine.

#### Type material.

The holotype, 9 paratype females and juveniles from all stages are deposited in the nematode collection of the Institute of Biodiversity and Ecosystem Research, Sofia, Bulgaria. Other paratypes deposited as follows: 15 females in the Crop Research Institute, Prague, the Czech Republic; 5 females in the USDA Nematode Collection, Beltsville, Maryland, USA; 5 females in the Nematode Collection of the Institute of Plant Protection, Bari, Italy; 5 females in the Wageningen Nematode Collection (WANECO), Wageningen, the Netherlands. The ribosomal and mtDNA sequences (18S rDNA, ITS1, ITS2, D2-D3, *cox*1, *nad*4) of *Xiphinema browni* sp. n. are deposited in GenBank (for accession numbers see Table 2).

#### Sequence and phylogenetic analyses.

There was no sequence variation between populations for 18S and D2-D3, ITS1 and ITS2 rDNA regions of *Xiphinema browni* sp. n. Of all four populations studied *cox*1 region of three population from the Czech Republic (Kurdějov, Mohyla Míru, Sokolnice) were sequenced by Kumari et al. (2010b) and all populations were identical therefore only one population was submitted to GenBank (accession number GU222424). The Slovakian population was sequenced in this study and it was identical to previously published sequence of Kurdějov the population identified as *Xiphinema pachtaicum* (GU222424, Kumari et al. 2010b). All four sequenced populations were also identical for *nad*4 part.

**Figure 9.**
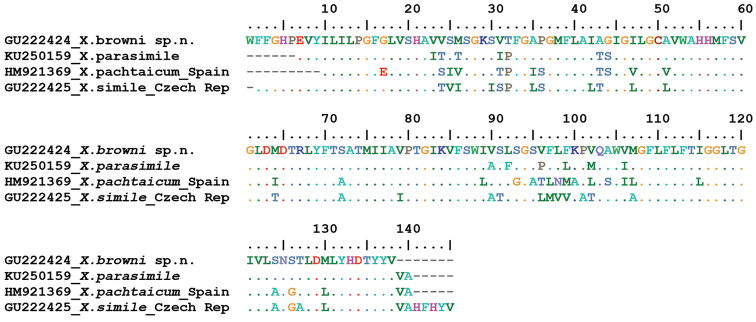
*Cox*1 amino acid sequence alignment of *Xiphinema browni* sp. n. and the closest species *Xiphinema parasimile*, *Xiphinema simile* and *Xiphinema pachtaicum*.

BLAST at NCBI using 18S and D2-D3 region sequences as queries revealed highest similarity (99 and 87%) to the corresponding sequences of *Xiphinema simile* Lamberti, Choleva & Agostinelli, 1983 from Serbia (AM086681) and two Spanish populations of *Xiphinema opisthohysterum* Siddiqi, 1961 (JQ990040 and KP268967), respectively. The estimated divergences (p-distance) between the 18S rDNA sequences of the new species and the closest species, *Xiphinema parasimile* from Bulgaria (this study) and *Xiphinema simile* from Serbia (AM086681) were 0.3 (6 nt) and 1.2% (21 nt), respectively. Again, the new D2-D3 sequence of *Xiphinema parasimile* from Bulgaria was most similar (p-distance = 4.6%), followed by the Serbian populations of *Xiphinema parasimile* (p-distance = 7.6–7.9%, calculated for D2 region only) and various populations of *Xiphinema simile* (14.1–14.7%). The partial *cox*1 sequences of *Xiphinema browni* sp. n revealed highest similarity to *Xiphinema simile* from Slovakia (AM086708). Surprisingly, these two species showed very high similarity 99% (2 nts difference) in *cox*1 sequences and higher dissimilarity in 18S rDNA (p-distance = 1.2%, 21 nts). Other authors (Gutiérrez-Gutiérrez et al. 2012) have also reported similar observation namely, 100% identity in *cox*1 part of two different species *Xiphinema duriense* Lamberti, Lemos, Agostinelli & D’Addabo, 1993 (JQ990053) and *Xiphinema opistohysterum* (JQ990054) and clear separation in D2-D3 28S sequences (or 96 % identity). Further, the *cox*1 sequences of *Xiphinema browni* sp. n. and the closest species *Xiphinema parasimile*, *Xiphinema simile* (GU222425, Czech Republic) and *Xiphinema pachtaicum* (HM921369, Spain) were translated to amino acids and aligned (Fig. 9). The estimated p-distances between *Xiphinema browni* sp. n. and the three species were 10.1%, 21.7% and 23.3%, respectively.

**Figure 10.**
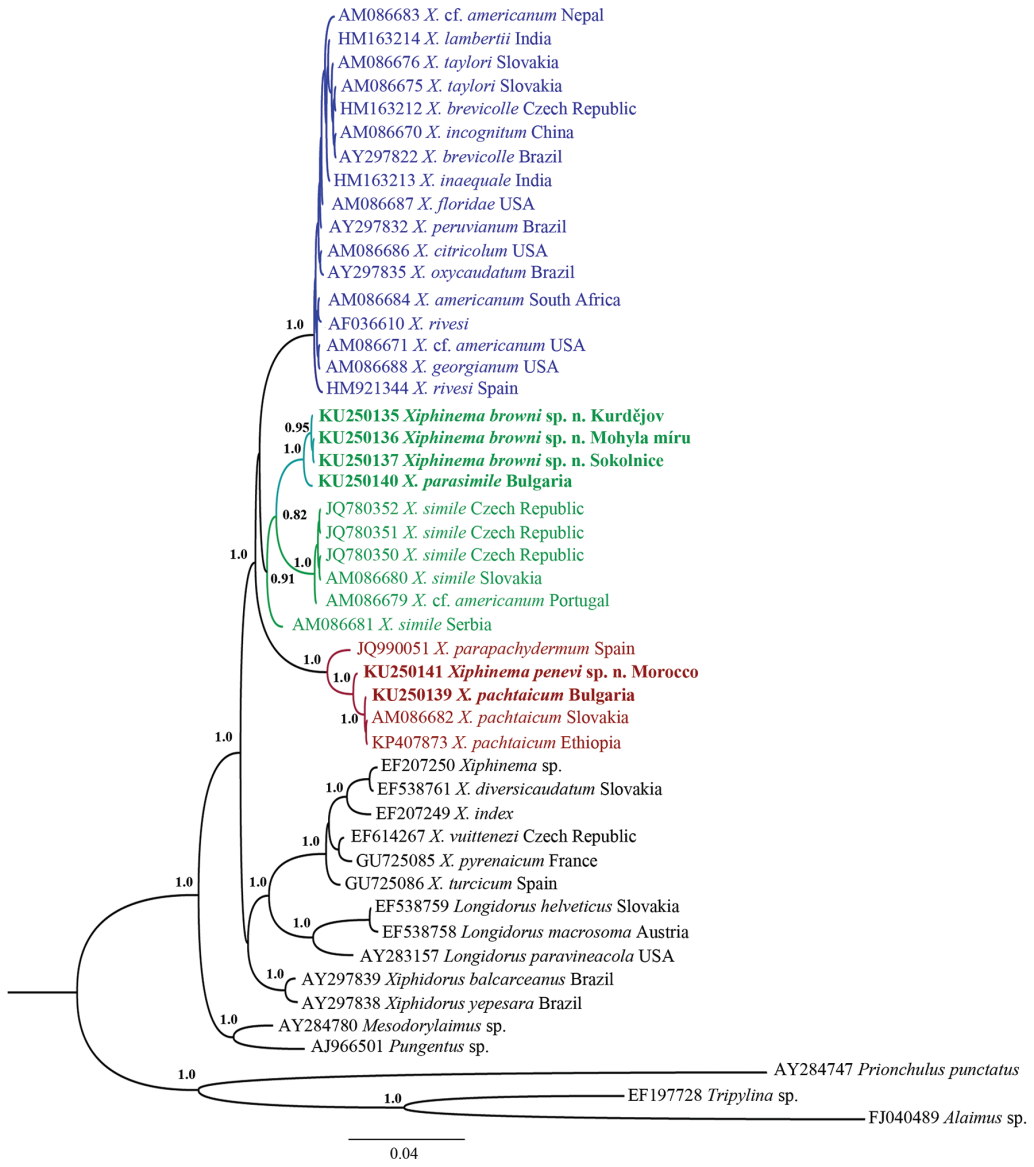
Hypothesis of the phylogenetic relationships of *Xiphinema browni* sp. n., *Xiphinema parasimile*, *Xiphinema pachtaicum* and *Xiphinema penevi* sp. n. based on 18S rDNA inferred from a Bayesian analysis using GTR+G model and *Prionchulus punctatus* (Cobb, 1917) Andrássy, 1958, *Alaimus* sp. and *Tripylina* sp. as an outgroup. Posterior probabilities higher than 0.8 are presented. The sequence of *Xiphinema browni* from Moča was not included due to the shorter length.

**Figure 11.**
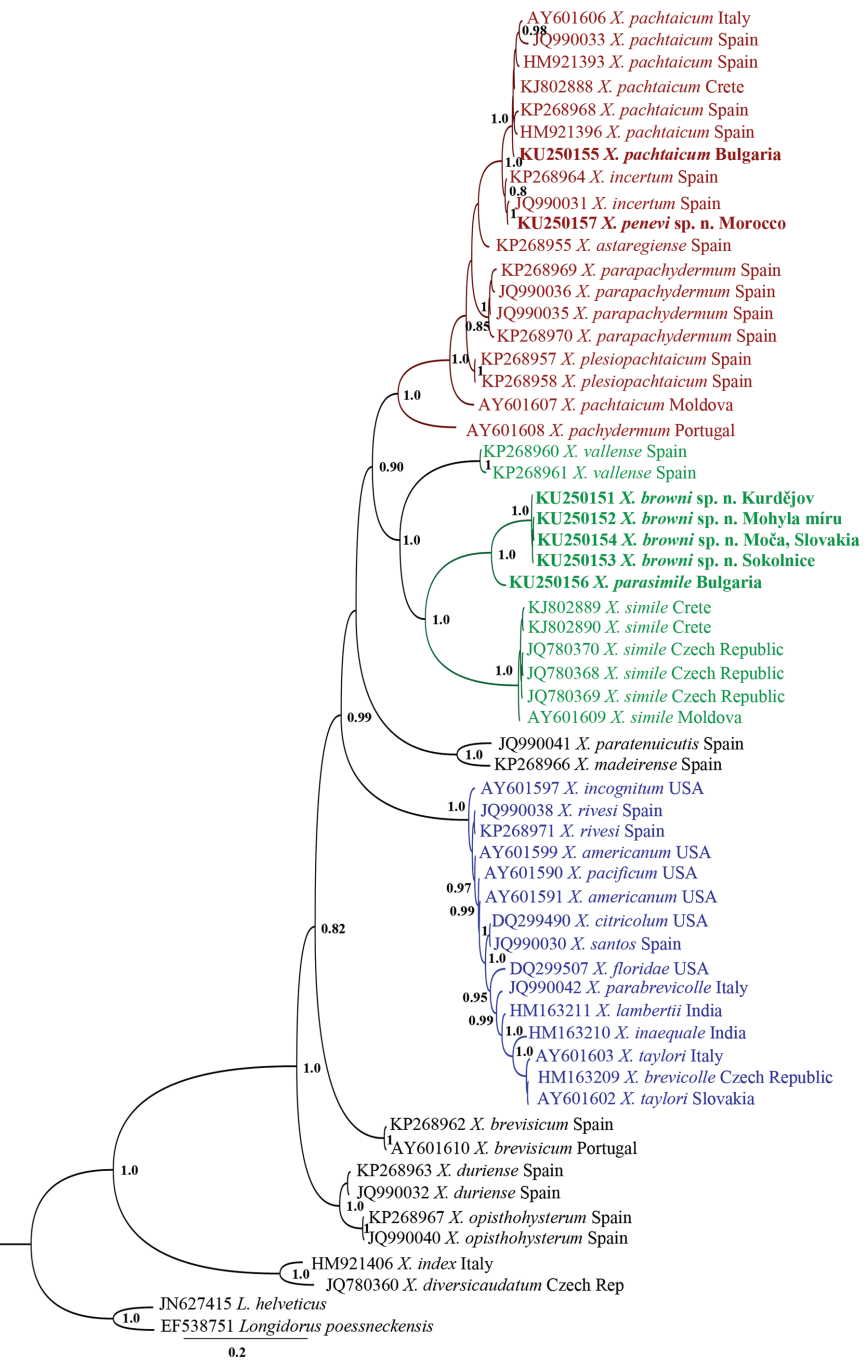
Hypothesis of the phylogenetic relationships of *Xiphinema browni* sp. n., *Xiphinema parasimile*, *Xiphinema pachtaicum* and *Xiphinema penevi* sp. n. based on 28S rDNA inferred from a Bayesian analysis using GTR+G model and *Longidorus helveticus* Lamberti, Kunz, Grunder, Molinari, De Luca, Agostinelli & Radicci, 2001 and *Longidorus poessneckensis* Altherr, 1974 as an outgroup. Posterior probabilities higher than 0.8 are presented.

**Figure 12.**
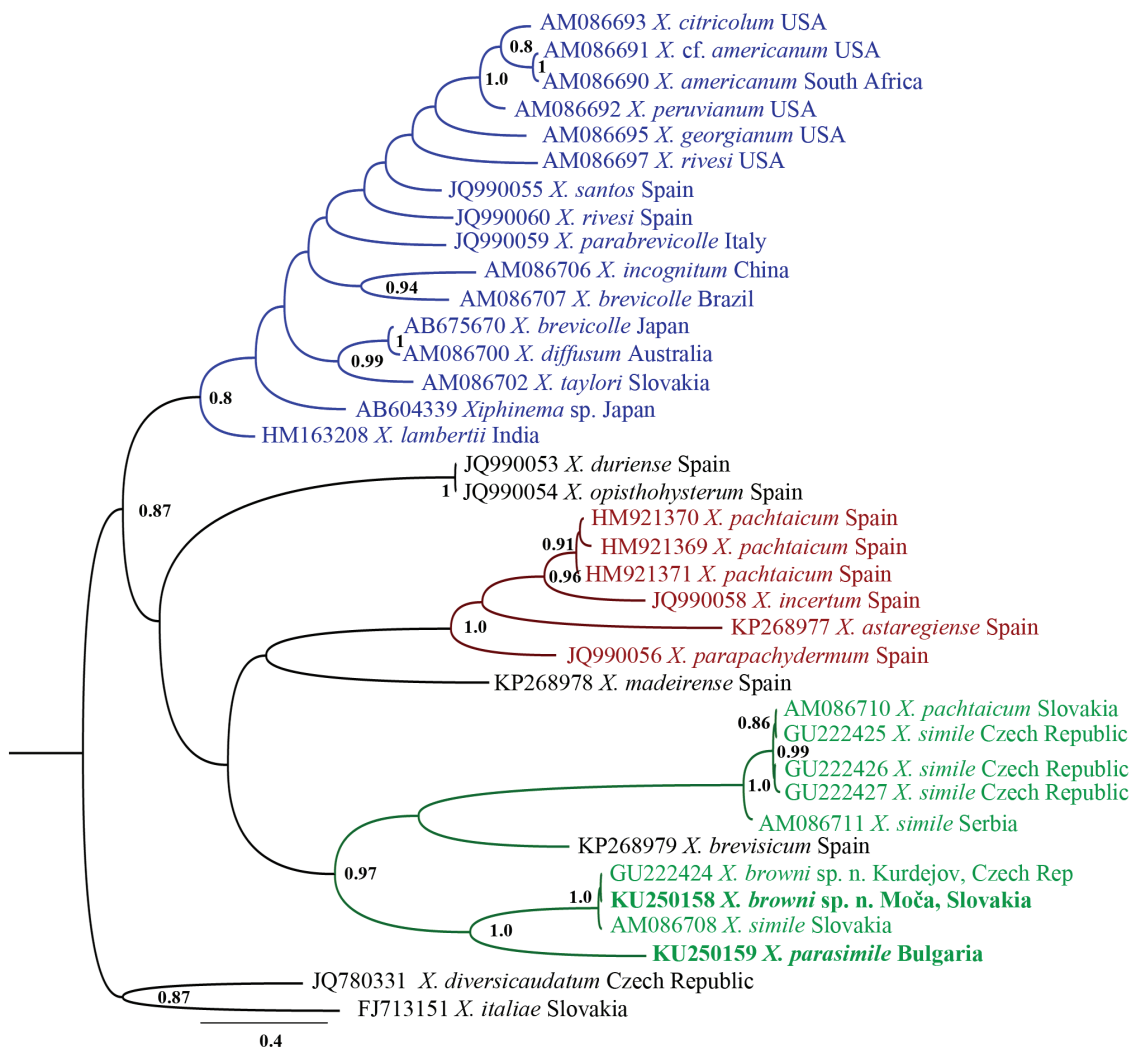
Hypothesis of the phylogenetic relationships of *Xiphinema browni* sp. n. and *Xiphinema parasimile* based on *cox*1 inferred from a Bayesian analysis using GTR+G model and *Xiphinema italiae* Mayl, 1953 and *Xiphinema diversicaudatum* (Micoletzky, 1927), Thorne, 1939 as an outgroup. Posterior probabilities higher than 0.8 are presented.

**Figure 13.**
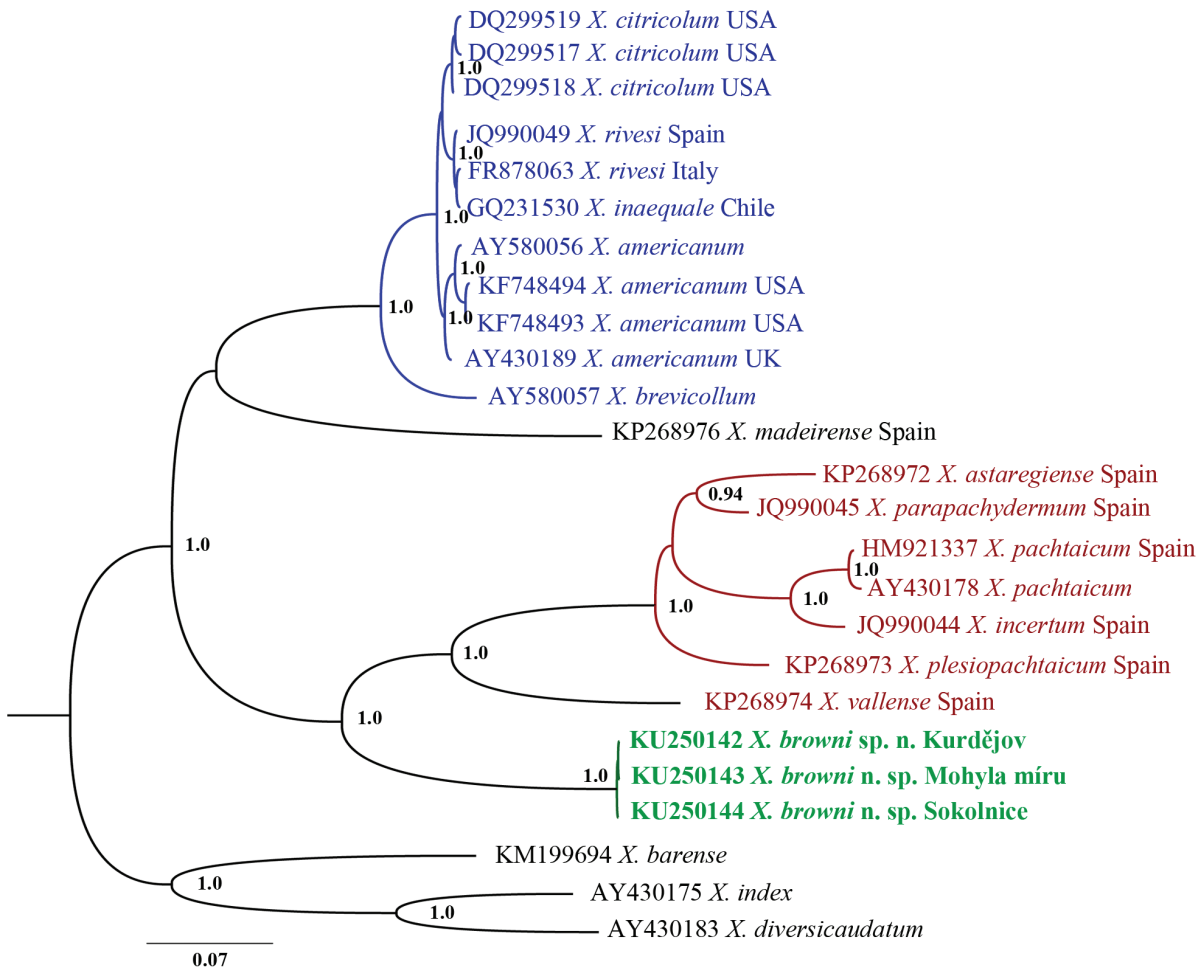
Hypothesis of the phylogenetic relationships of *Xiphinema browni* sp. n. based on ITS1 inferred from a Bayesian analysis using GTR+G model and *Xiphinema barense* Lamberti, Roca, Agostinelli, Bleve-Zacheo, 1986, *Xiphinema italiae* and *Xiphinema diversicaudatum* as an outgroup. Posterior probabilities higher than 0.8 are presented.

**Figure 14.**
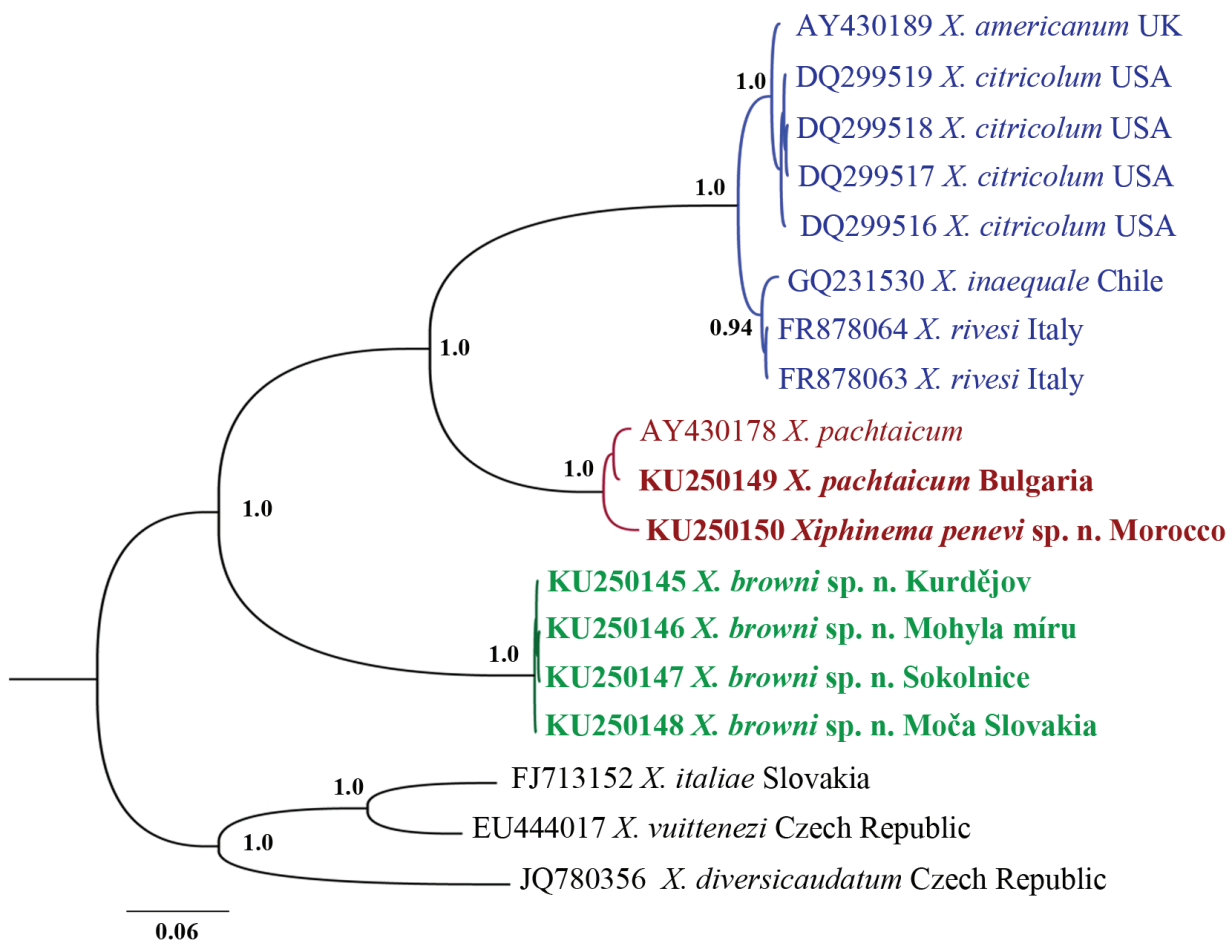
Hypothesis of the phylogenetic relationships of *Xiphinema browni* sp. n., *Xiphinema parasimile*, *Xiphinema pachtaicum* and *Xiphinema penevi* sp. n. based on ITS2 inferred from a Bayesian analysis using GTR+G model and *Xiphinema italiae*, *Xiphinema diversicaudatum* and *Xiphinema vuittenezi* Luc, Lima, Weischer & Flegg, 1964 as an outgroup. Posterior probabilities higher than 0.8 are presented.

In all three phylogeny reconstructions (18S, D2-D3 and *cox*1) *Xiphinema parasimile* from Bulgaria was a sister species of *Xiphinema browni* sp. n. and both species were part of a well supported clade with other European populations of *Xiphinema simile* (Figs 10–12). The recently described species *Xiphinema vallense* Archidona-Yuste, Navas-Cortes, Cantalapiedra-Navarrete, Palomares-Rius & Castillo, 2016 presented only with D2-D3 and ITS1 rDNA sequences seems also to be evolutionary very closely related (Figs 11 and 13), however amplifying additional sequences for other molecular markers (e.g. 18S and *cox*1) could help to better clarify its relationships. The position of the new species in the phylogeny trees based on ITS1 and ITS2 sequences was unstable (Figs 13 and 14). The analyses resulted in various tree topologies when using different alignment algorithms and reconstruction methods (ML and BI) and because of the absence of homologous sequences from closely related species. In most cases *Xiphinema browni* sp. n. was part of a clade of European *Xiphinema americanum*-group species considered as group II in a previous publication (Archidona-Yuste et al. 2016). Due to insufficient number of *nad*4 sequences of species belonging to the *Xiphinema americanum*-group at NCBI no phylogenetic reconstructions are presented.

**Diagnosis and relationships.** *Xiphinema browni* sp. n. is characterised by a unique combination of traits: slender and medium sized body (1.6–2.41 mm) and odontostyle (73–85 μm), lip region expanded, laterally rounded, separated from the rest of body by a constriction, post-equatorial vulva position (V=52–58 %), symbiotic bacteria present, female tail conical dorsally convex, with narrow rounded to pointed tip, 24–35 μm long, (c=53.4–86.8; c’=1.5–2.1), and specific ribosomal and mtDNA sequences (Table 2). The alpha-numeric codes based on average values (ranges given in parentheses) using the polytomous key by Lamberti et al. (2004) are: A3 (2), B3 (2), C3 (4), D2 (1/3), E2 (3), F2 (1/3), G2, H1, I2 (1/3).

Species having similar morphometrics to *Xiphinema browni* sp. n. based on type populations are presented in Table 6. Recently described species *Xiphinema parasimile*, *Xiphinema parabrevicolle* Gutiérrez-Gutiérrez, Cantalapiedra-Navarrete, Decraemer, Vovlas, Prior, Palomares-Rius & Castillo, 2012, *Xiphinema parapachydermum* Gutiérrez-Gutiérrez, Cantalapiedra-Navarrete, Decraemer, Vovlas, Prior, Palomares-Rius & Castillo, 2012, *Xiphinema paratenuicutis* Gutiérrez-Gutiérrez, Cantalapiedra-Navarrete, Decraemer, Vovlas, Prior, Palomares-Rius & Castillo, 2012, *Xiphinema plesiopachtaicum* Archidona-Yuste, Navas-Cortes, Cantalapiedra-Navarrete, Palomares-Rius & Castillo, 2016 and *Xiphinema vallense* (Barsi and Lamberti 2004, Gutiérrez-Gutiérrez et al. 2012, Archidona-Yuste et al. 2016) have also been compared. Six of these species have non-European distribution (Table 6) whereas the others were described from and/or found mainly in Europe. *Xiphinema simile* was also included in the table comparing morphometrical data because of the close relationships based on sequence and phylogenetic analyses and its wide distribution in many European countries.

**Table 6.** Morphometric data of *Xiphinema americanum* species having similar morphometrics with the new species based on type populations.

	**Non-European species**							**European species**
	*Xiphinema penevi* sp. n.	*Xiphinema bricolensis*	*Xiphinema californicum*	*Xiphinema citricolum*	*Xiphinema intermedium*	*Xiphinema oxycaudatum*	*Xiphinema tenuicutis*	*Xiphinema plesiopachtaicum*
Body L	1.69 (1.5–1.85)	1.9 (1.7–2.3)	2 (1.8–2.2)	1.6–1.8	1.6 (1.4–1.9)	1.6 (1.5–1.7)	1.8 (1.6–1.9)	1.9 (1.5–2.1)
a	61 (57.2–65.0)	56 (52–62)	60 (52–68)	45–46	43 (38–51)	47 (45–51)	46 (40–53)	64 (57.3–70.2)
c	57.7 (50.8–61.5)	57 (49–65)	63 (58–76)	44–50	47 (41–59)	51 (48–54)	61 (56–65)	71.1 (62.5–88.7)
c’	1.8 (1.6–1.9)	1.5 (1.3–1.6)	1.6 (1.3–1.9)	1.6–1.7	1.5 (1.3–1.7)	1.6 (1.3–1.7)	1.5 (1.4–1.7)	1.4 (1.3–1.7)
Vulva [%]	57 (51–61.5)	52 (50–55)	51 (49–55)	52–54	52 (50–57)	52.5 (51–54)	51 (47–52)	57.3 (55.5–60)
Odontostyle L	77 (72–79)	87 (85–94)	90 (83–98)	78–86	76 (68–80)	82 (78–84)	76 (73–80)	83 (77–89)
Tail L	29 (26–32)	36 (31–41)	31 (27–36)	34–36	33 (31–38)	33 (27–35)	29 (26–32)	26 (23–28)
Length to GR	68 (66–71)	68 (61–76)	76 (66–83)	64–72	63 (58–67)	71 (66–75)	60 (55–64)	69 (63–76.5)
Lips width	8 (8–9)	11	10 (10–11)	12.5	10.5 (9.5–11)	10 (9–10)	9 (9–10)	9.5 (8.5–10.5)
J	9 (8–10)	(6–7)	6 (5–8.5)	12–14	10 (9–12)	9 (7–10)	8 (6.5–10)	8 (5.5–10)
Juvenile stages	4	?	4	3		?		?
Males (number of VM supplements)	rare or absent	rare or absent 11	rare or absent 7	rare or absent 10	rare or absent 11	rare or absent 3	not found	Not found
	**European species**							
	*Xiphinema browni* sp. n.	*Xiphinema microstilum*	*Xiphinema pachtaicum*	*Xiphinema parasimile*	*Xiphinema paratenuicutis*	*Xiphinema simile*	*Xiphinema parapachydermum*	*Xiphinema vallense*
Body L	2.03 (1.8–2.40)	2.6 (2.5–2.8)	1.88	1.99 (1.75–2.26)	2.01 (1.7–2.2)	1.9 (1.7–2.1)	1.78 (1.41–2.0)	2.0 (1.8–2.2)
a	69.3 (56.9–81.3)	86 (77–93)		70.5 (61.0–76.1)	61.1 (51.9–69.7)	71 (63–77)	64 (51.3–73.1)	68.9 (61.6–79.1)
c	69.9 (54.7–83.0)	74 (63–88)	72.3	59.9 (50.9–69.8)	68.8 (58.8–79.9)	67 (61–70)	60.3 (46.3–75.5)	73.4 (58.2–86.3)
c’	1.8 (1.53–2.07)	1.8 (1.6–2.0)	1.6	2.02 (1.79–2.28)	1.4 (1.2–1.6)	1.7 (1.6–1.8)	1.8 (1.5–2.3)	1.6 (1.4–1.7)
Vulva [%]	55 (52.3–58.5)	57 (55–60)	60	55.5 (52.2–58.7)	56.8 (55–60)	53 (51–54)	59 (55–66)	57.5 (55–59.5)
Odontostyle L	79 (75–83)	74 (68–77)	83	69.7 (64.4–73.7)	75.2(71.5–83)	66 (62–69)	81 (70–87.5)	79 (73–85.5)
Tail L	29 (25–33)	35 (31–39)	26	33.3 (30.3–37.1)	29.4 (25–34.5)	29 (27–30)	28.8 (26.5–35.5)	27.8 (22.5–34)
Length to GR	71 (65–75)	63 (57–68)	78	62.6 (59.4–66.3)	63.2 (60–69)	51 (49–53)	70 (59.5–75.5)	69.5 (62–72.5)
Lips width	8 (8–10)	9 (9–10)	10	9.0 (8.4–9.7)	9.6 (9–10)	9 (9–9)	8.8 (8–9.5)	8.5 (8–9)
J	8 (6–12)	10 (7–12)		8.2 (6- 10)	8.4 (6.5–10.0)	7 (6–8)	9.3 (7–12.5)	7.6 (6.5–8.5)
Juvenile stages	4		4	4	4	3	4	4
Males (number of supplements)	rare or absent 5	frequent 4–5	rare or absent 5–6	rare or absent 5	males abundant 5	rare or absent 3–5	Males abundant	Rare or absent 6, 7

Based both on morphology and molecular data *Xiphinema browni* sp. n. is most similar with *Xiphinema parasimile*, *Xiphinema simile* and *Xiphinema vallense*. Morphologically, it can be distinguished from:

*Xiphinema parasimile* by its different lip region shape (expanded *vs* not expanded), somewhat longer odontostyle av. 79–83 (73–85) μm *vs* av. 70 (64–74) μm in the type population, avs. 69–70 (63–74) in Bulgarian populations and avs. 68–70 (67–72) μm in females from Romania (Barsi and Lamberti 2004, Lazarova et al. 2008, Bontă et al. 2012);

*Xiphinema simile* by its longer odontostyle av. 79–83 (73–85) *vs* av. 66 (62–69) in type population, avs. 68.5–70 (66–72.5) in other Bulgarian populations, 67.5 (65–70) μm in a population from Bosna and Herzegovina, and avs. 67–68 (61–73) μm in females from the Czech Republic (Lamberti et al. 1983, Barsi and Lamberti 2004, Kumari 2006, Lazarova et al. 2008). However, it should be noted that females from Serbia and Crete (odontostyle 71.5 (66–74) μm and 75–77 μm, respectively) have slightly overlapping values between *Xiphinema browni* sp. n. and *Xiphinema simile* for this character (Barsi and Lamberti 2004, Tzortzakakis et al. 2014). Further *Xiphinema browni* sp. n. differs from *Xiphinema simile* in the length and structure of uteri (in the new species separate uteri and ovejector present *vs* separate uteri not present), different tail shape (conoid *vs* bluntly conoid), and in the shorter bulbus (53–69 *vs* 76–92 μm) (Lazarova et al. 2008) (Table 4). Finally, *Xiphinema browni* sp. n. develops though 4 *vs* 3 juvenile stages in *Xiphinema simile*;

*Xiphinema vallense* by the position of amphideal fovea aperture (posterior constriction level *vs* on the lips); higher lip region (4–7 μm *vs* 2–3.5 μm); presence of symbiont bacteria in ovaries *vs* ovaries without symbionts; somewhat higher c’ values (c’=1.8 (1.53–2.07) *vs* c’=1.6 (1.4–1.7); the different tail shape (dorsoventral depression at hyaline region level not present *vs* present); shorter spicules in males (29 μm *vs* 38 μm).

**Figure 15.**
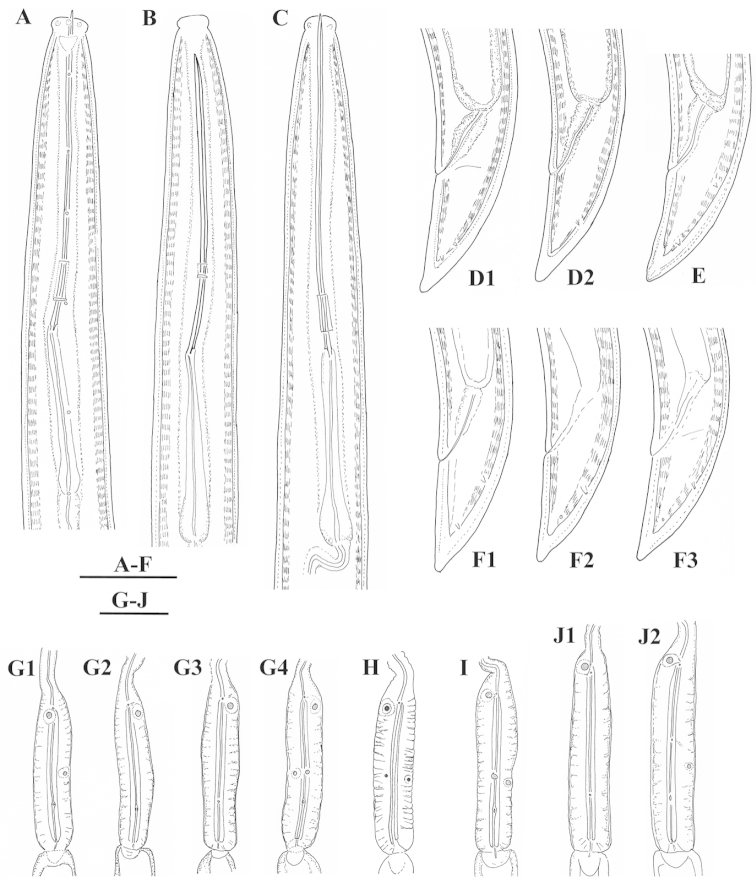
*Xiphinema browni* sp. n., *Xiphinema penevi* sp. n. *Xiphinema pachtaicum* and *Xiphinema parasimile*. Female: **A–C** Anterior ends **D–F** Tail shapes **G–J** Pharyngeal bulbs **A, D, G** *Xiphinema browni* sp. n. **B, E, I** *Xiphinema penevi* sp. n. **C, F, J** *Xiphinema pachtaicum* **H** *Xiphinema parasimile*. Scale bars: 25 μm

**Figure 16.**
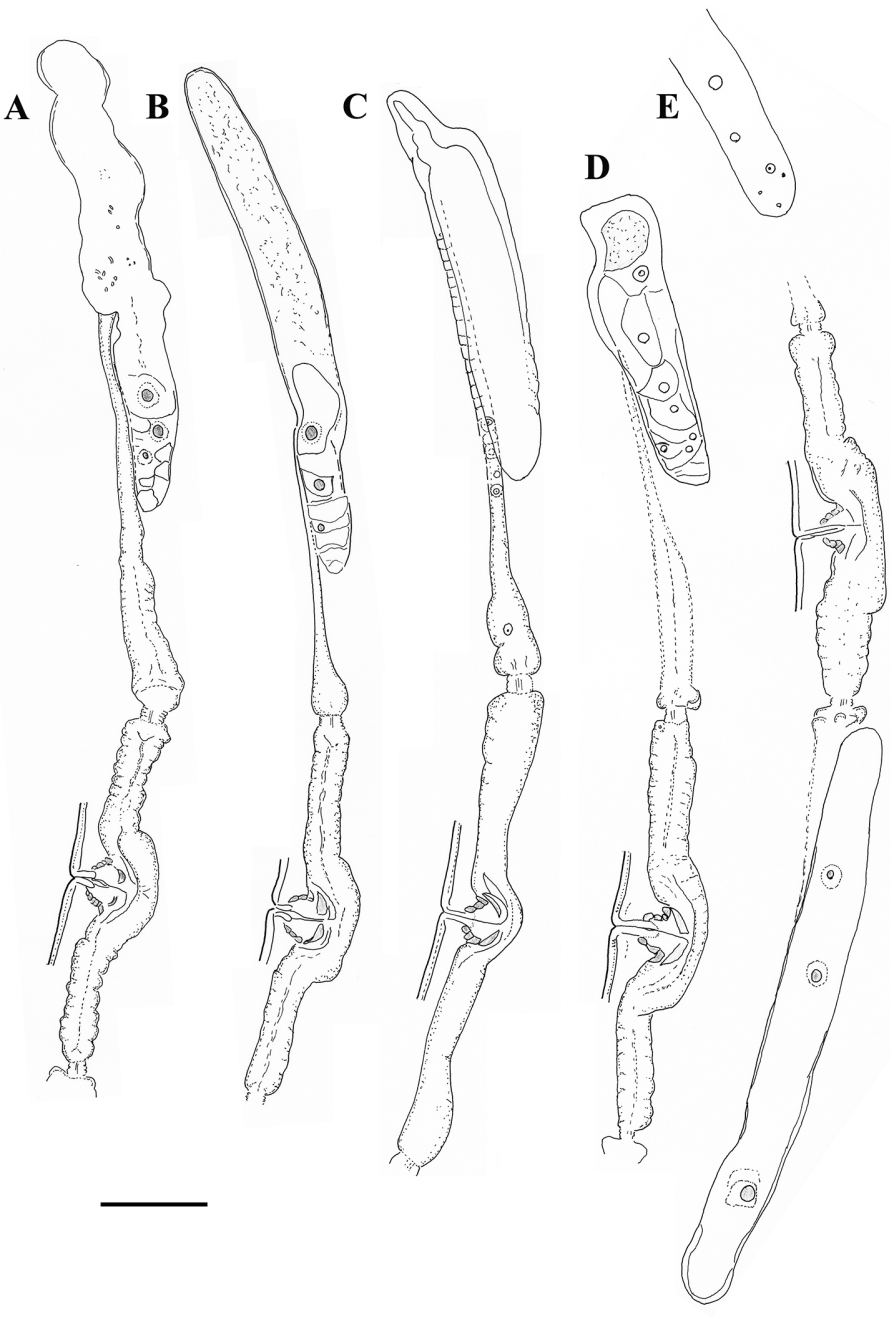
*Xiphinema browni* sp. n., *Xiphinema penevi* sp. n. and *Xiphinema pachtaicum*. Female genital system comparison: **A, E** Posterior genital branch **B–D** Anterior genital branch **A, B** *Xiphinema browni* sp. n. **C** *Xiphinema penevi* sp. n. **D, E** *Xiphinema pachtaicum*. Scale bars: 25 μm

**Figure 17.**
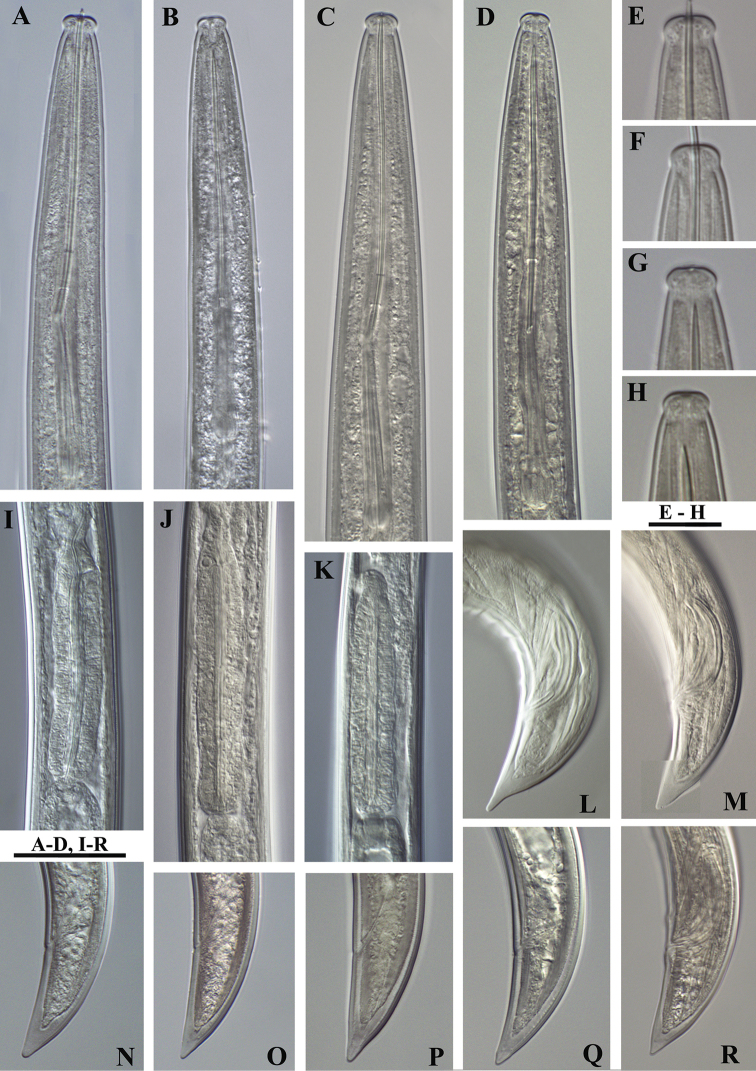
*Xiphinema browni* sp. n., *Xiphinema parasimile*, *Xiphinema pachtaicum* and *Xiphinema penevi* sp. n. Female and male: **A–D** Anterior ends **E–H** Labial region **I–K** Pharyngeal bulbs **L, M, R** Male tails **N–Q** Female tails **A, E, I, M, N** *Xiphinema browni* sp. n. **B, F, K, O, R** *Xiphinema parasimile* **C, G, J, L, P** *Xiphinema pachtaicum* **D, H, Q** *Xiphinema penevi* sp. n. Scale bars: 30 μm **(A–D, I–R)**; 12 μm **(E–H)**.

**Figure 18.**
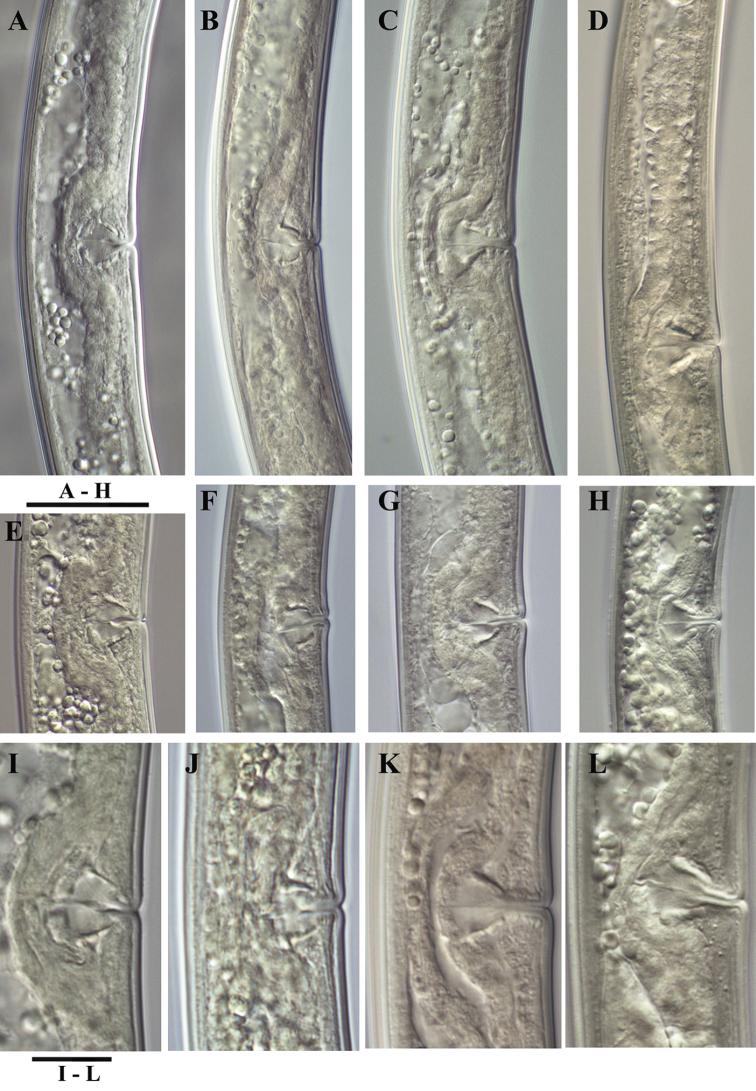
*Xiphinema browni* sp. n., *Xiphinema parasimile*, *Xiphinema pachtaicum* and *Xiphinema penevi* sp. n. Female: **A–D** Genital system **E–L** Vulval region **A, E, I** *Xiphinema browni* sp. n. **B, F, J** *Xiphinema parasimile* **C, D, G, K** *Xiphinema pachtaicum* **H, L** *Xiphinema penevi* sp. n. Scale bars: 30 μm **(A–H)**; 12 μm **(I–L)**.

Additionally, *Xiphinema browni* sp. n. can be differentiated from:

*Xiphinema pachtaicum* by the different vagina shape (bell-shaped *vs* funnel shaped, (Figs 16, 18) and shorter *pars distalis vaginae*, shorter pharyngeal bulb (53–69 *vs* 75–80 μm), more posterior location of the dorsal nucleus (DN=13–21% *vs* 9–10 %) (Table 4), different tail shape in both sexes (conical *vs* subdigitate). Illustrations of selected features of the closest species *Xiphinema pachtaicum*, *Xiphinema parasimile* and *Xiphinema penevi* sp. n. are presented in Figs 15–18 for comparison.

*Xiphinema paratenuicutis* in having symbionts in its ovaries *vs* absent, males rare *vs* abundant, higher values for c’ (1.8 (1.5–2.1) *vs* 1.4 (1.2–1.6), different location of dorsal nucleus (DN after beginning of the stronger cuticular lining of the bulbus *vs* before, see Fig. 1 D1-F and Fig. 2E in Gutiérrez- Gutiérrez et al. 2012);

*Xiphinema plesiopachtaicum* by the position of the amphideal aperture (posterior *vs* at the constriction level); somewhat shorter bulbus (avs. 59–60 (53–69) *vs* av. 73 (60–86); shorter uteri (av. 81 *vs* av. 138 μm); higher c’ values (c’=1.8 (1.53–2.07) *vs* c’=1.4 (1.3–1.7); and differently shaped vagina (bell-shaped *vs* funnel shaped).

For comparison between *Xiphinema browni* sp. n. and *Xiphinema penevi* sp. n. see below.

#### Etymology.

The species is named after Prof Derek JF Brown, an outstanding nematologist, for his significant contributions to the knowledge of plant parasitic nematodes and the development of nematology in Bulgaria.

### Xiphinema penevi sp. n.

Taxon classificationAnimaliaDorylaimidaLongidoridae

http://zoobank.org/C98CE5B3-9BAE-423C-B887-9BFFFD489798

Figures 15 , 16 , 17 , 18 , 19 , 20 , 21 , 22 , 23

#### Measurements.

See Tables 4, 5, 7.

**Table 7.** Morphometrics of *Xiphinema penevi* sp. n. (females and juveniles) from *Quercus ilex* Morocco. All measurements except ratios in micrometres given as mean ± standard deviation (range).

Characters		Females	Juveniles			
	Holotype	Paratypes	J1	J2	J3	J4
n		12	2	8	5	5
L	1726	1687±100 (1532–1846)	664, 602	777.6±37 (702–816)	1049.0±54 (988–1126)	1318±38 (1292–1384)
a	62.1	61.0±2.6 (57.2–65.0)	40.6, 38.0	43.1±2.4 (40.2–47.0)	48.2±1.5 (46.7–49.8)	54.4±3.6 (51–58)
b	5.8	6.1±1.1 (5.0-7.0)	3.8, 3.6	4.0±0.2 (3.6–4.2)	4.5±0.1 (4.4–4.6)	5, 6
c	55.1	57.7±3.9 (50.8–61.5)	20.9, 22.4	24.7±2.5 (21.2–28.1)	33.4, 34.1	40.8±2.6 (38–43)
c’	2.0	1.8±0.1 (1.6–1.9)	2.9, 2.6	2.7±0.3 (2.4–3.1)	2.2, 2.3	2.1±0.2 (2–2)
V (%)	56.8	57.1±0.6 (55.9–58.1)				
G1(%)		11.2±0.5 (10.9–12.1)				
G2(%)		12.3±3.2 (9.2–19.5)				
Odontostyle	75	76.7±2.1 (72-79)	36.5, 37	43.8±1.0 (43–45)	54.5±1.1 (53–56)	63.3±2.0 (60–65)
Replacement odontostyle			43, 46	56.5±1.8 (53–58)	66.2±1.4 (65–68)	75.0±1.6 (73–78)
Odontophore	50	47.7±1.8 (44–50)	28	33.6±1.2 (32–35)	37.6±1.9 (35–39)	43.9±1.6 (42–45)
Oral aperture to guide ring	71	68.0±**0**.6 (66–71)	30.5, 33	38.5±1.2 (36–40)	49.0±1.4 (47–50)	55.6±3.9 (50–60)
Tail length	31	29.3±1.9 (26–32)	32, 27	32.0±2.7 (29–35.5)	32, 33	31.9±2.0 (30–34)
Length of hyaline part	9	8.4±0.7 (8–10)	4, 4	4.3±0.7 (3–5)	6, 6	6.9±0.7 (6–7)
Body diam. at: - lip region	9	8.3±0.3 (8–9)	7, 7	7.1±0.5 (6–8)	7.3±0.7 (7–8)	7.8±0.2 (8–8)
- at guiding ring	21	20.6±0.5 (20–21)	12, 13	14.5±0.6 (14–15)	16.2±0.7 (15–17)	18.6±0.9 (18–20)
- at base of pharynx	25	24.0±1.0 (22–26)	15, 15	16.8±1.2 (15–18)	19.3±0.8 (18–20)	22.3±1.0 (21–23)
- at mid body/at vulva	28	27.6±1.4 (25–31)	16, 16	18.1±1.5 (16–20)	21.8±1.3 (21–24)	24.0±1.7 (22–26)
- at anus	16	16.2±0.7 (15–17)	11, 10	11.9±0.8 (11–13)	25.2±22.1 (13.5–58.4)	15.4±0.5 (15–16)
- at beginning of hyaline part	7	7.1±0.4 (7–8)	5, 4	4.2±0.3 (4–5)	5, 5	6.5±0.5 (6–7)

#### Description.

*Females*. Body open spiral to C shaped. Thickness of the cuticle at postlabial region 1 μm, 1–1.5 μm at mid-body and 2–2.5 μm at post-anal region, outer cuticle layer not reaching the tail end. Labial region flat anteriorly, laterally rounded, set off from the rest of the body by constriction, 2.5–4 μm high. Amphideal fovea hardly visible, its opening 4 μm in a paratype specimen (40–47 % of the corresponding body width); Distance between first and second guide ring in specimens with retracted odonostyle, 2.5–5 μm long. Odontophore with well developed flanges, 6–9 μm wide, often a small vestigium located in odonthophore area. Pharyngeal characters presented at Table 4. Dorsal nucleus 2.5–3 μm diam., ventrosublateral nuclei well visible, 2–2.5 μm. Prerectum indistinct, rectum 21.6±1.8 (19–24) μm, n=8, c 1.3 of corresponding body width. Reproductive system amphidelphic, symbiotic bacteria present in the ovaries. Uteri short, ovejector not developed, only in one specimen a structure resembling ovejector was observed (Table 5); vagina c. 2/3 of the corresponding body width, *pars proximalis vaginae* with well developed wall. Tail conoid, dorsally convex, ventrally slightly concave, gradually narrowing to a pointed tip, two distinct pairs of caudal pores.

*Male*. Not found.

*Juveniles*. The scatter diagram based on functional and replacement odontostyle, and body length revealed the presence of four juvenile stages (Fig. 23). As in most species of the *Xiphinema americanum*-group there is a gradually decreasing of **c**‘ values with successive stages which reflects increasing body width while the tail length is more or less similar in juveniles and adults.

**Figure 19.**
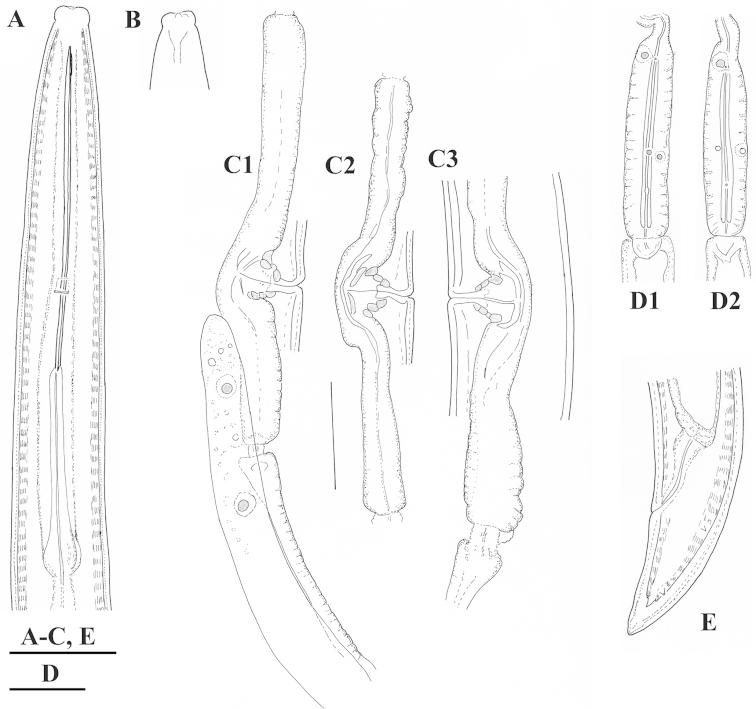
*Xiphinema penevi* sp. n. Female. **A** Anterior end **B** Amphideal fovea outline **C** Variations in genital system: **C1** Anterior uterus and partim posterior genital branch **C2, C3** Region of vagina and uteri **D** Pharyngeal bulb **E** Tail. Scale bars: 25 μm.

**Figure 20.**
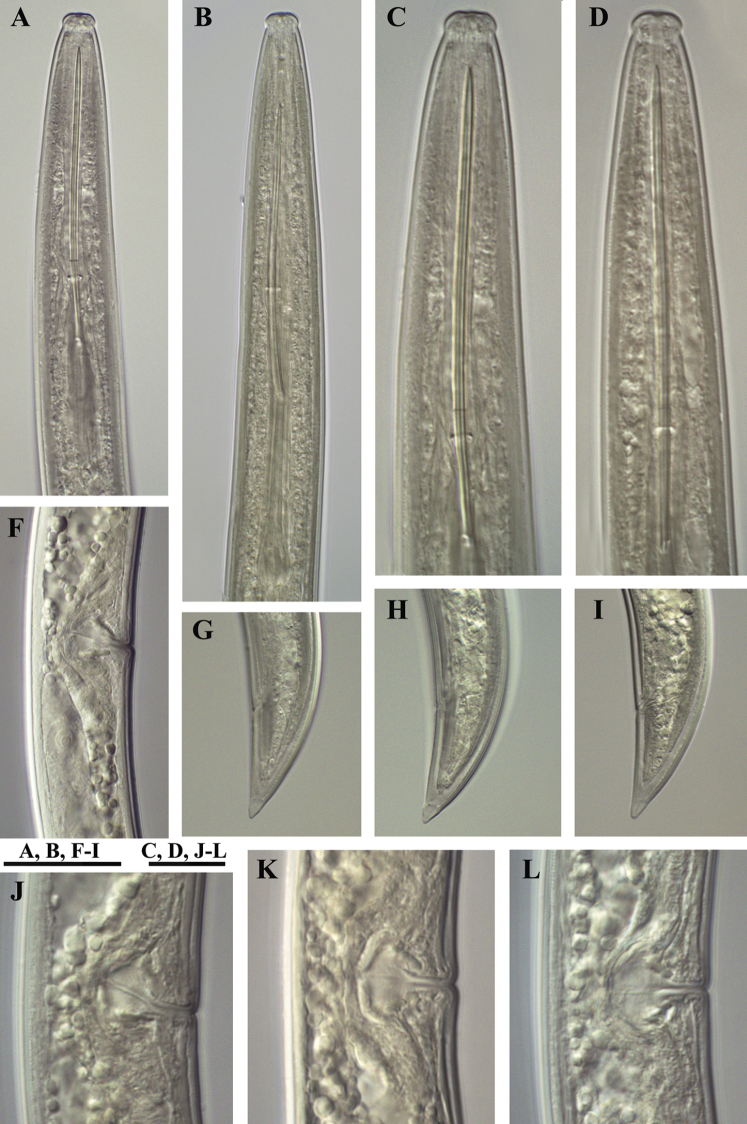
*Xiphinema penevi* sp. n. Female. Variations in: **A–D** Anterior ends (B-holotype) **F, J–L** Vagina region **G–I** Tail shapes. Scale bars: 30 μm **(A, B, F–I)**; 12 μm **(C, D, J–L)**.

**Figure 21.**
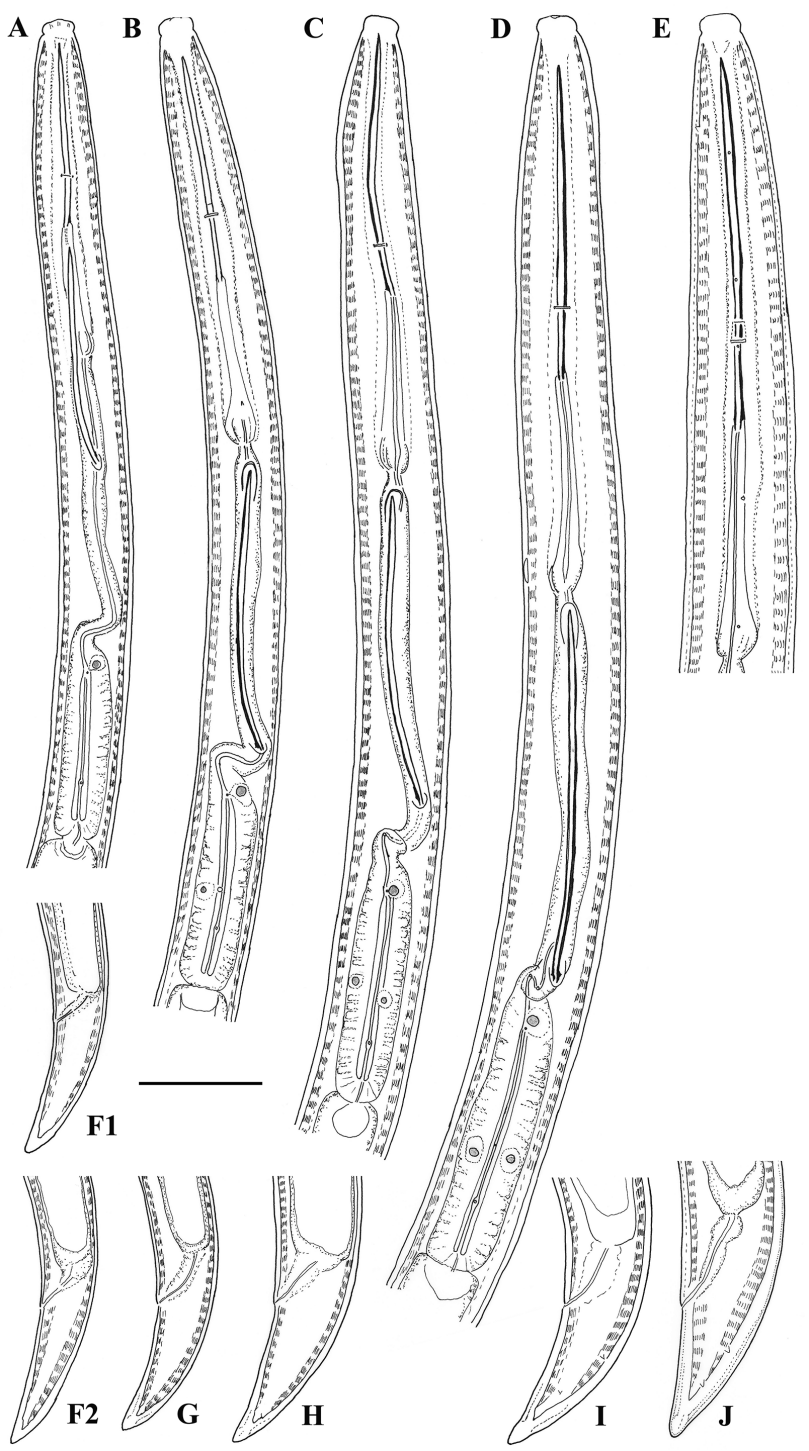
*Xiphinema penevi* sp. n. *Juveniles*: **A–E** Anterior ends of first- to fourth-stage juveniles and female **F–J** Tails of first to fourth juvenile stages and female (**F1** and **F2** tail of first stage juveniles). Scale bar: 25 μm.

**Figure 22.**
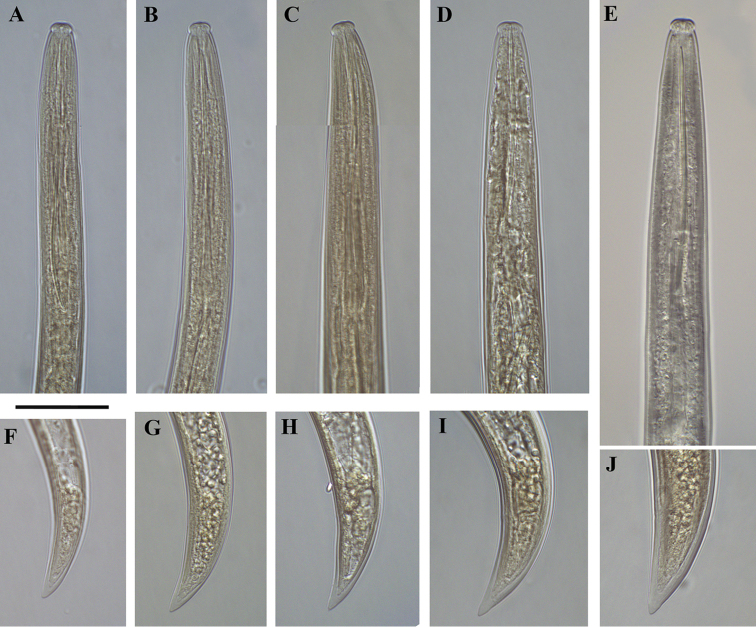
*Xiphinema penevi* sp. n. *Juveniles and female*: **A–D** Neck region of first- to fourth-stage juveniles **E** Anterior end of female **F–J** Tails of first to fourth juvenile stages and female. Scale bar: 30μm.

**Figure 23.**
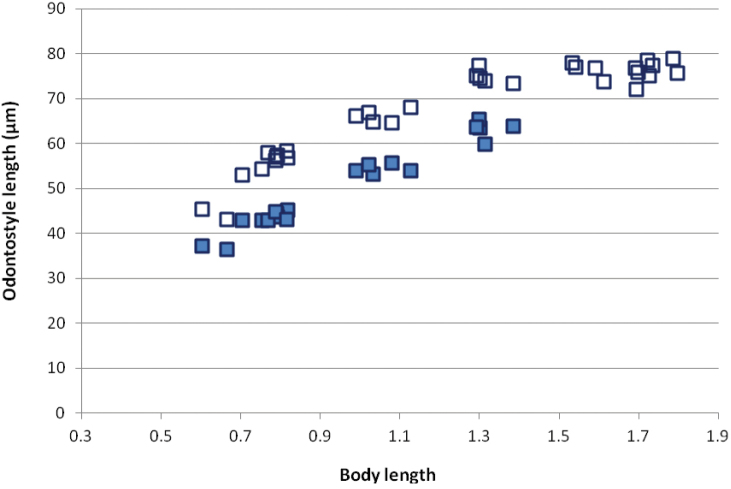
Scatter plot of odontostyle (■) and replacement odontostyle (□) against body length of *Xiphinema penevi* sp. n. juveniles and females from Morocco.

**Figure 24.**
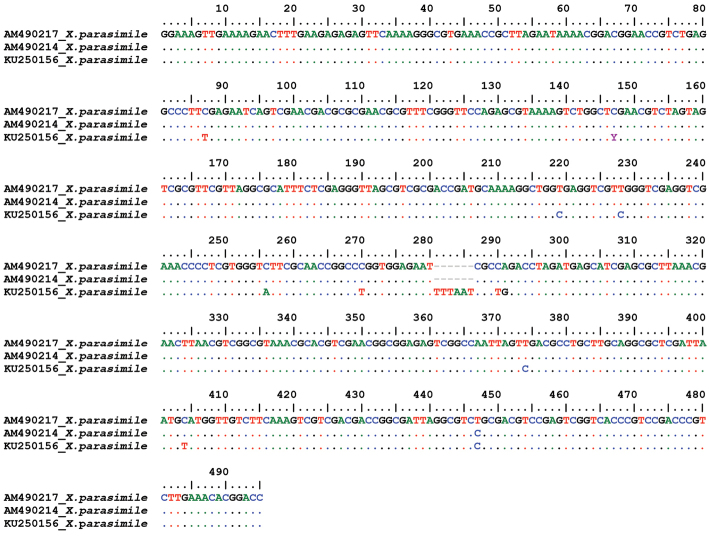
Sequence alignment of D2 28S rDNA region of *Xiphinema parasimile* from Bulgaria (KU250156) and Serbia (AM490214 and AM490217).

#### Type locality and plant association.

Ifrane, Morocco, *Quercus ilex* L. forest.

#### Type material.

The holotype, 7 paratype females and juveniles from all stages are deposited in the nematode collection of the Institute of Biodiversity and Ecosystem Research, Sofia, Bulgaria. Other paratypes deposited as follows: 2 females in the USDA Nematode Collection, Beltsville, Maryland, USA; 2 females in the Nematode Collection of the Institute of Plant Protection, Bari, Italy; 1 female in the Wageningen Nematode Collection (WANECO), Wageningen, the Netherlands. Three ribosomal sequences (18S, ITS2 and D2-D3) of *Xiphinema penevi* sp. n. are deposited in GenBank (for accession numbers see Table 2).

#### Sequence and phylogenetic analyses.

Sequences for three gene regions were obtained (18S, D2-D3 and ITS2). BLAST at NCBI using any of these sequences as queries revealed highest similarity to *Xiphinema pachtaicum* (99% for 18S, 6 nt difference), two populations of *Xiphinema incertum* Lamberti, Choleva & Agostinelli, 1983 from Spain (99% for D2-D3, 1 and 3 nt difference) and *Xiphinema pachtaicum* (90% for ITS2). In both 18S and D2-D3 phylogeny reconstructions *Xiphinema penevi* sp. n. was part of well supported clades with other species of *Xiphinema pachtaicum*-subgroup (*Xiphinema pachtaicum*, *Xiphinema parapachydermum* for 18S and *Xiphinema incertum*, *Xiphinema pachtaicum*, *Xiphinema parapachydermum*, *Xiphinema plesiopachtaicum*, *Xiphinema pachydermum* Sturhan, 1983 for D2-D3). In the phylogeny reconstruction based on ITS2 sequences, the species grouped with two other *Xiphinema pachtaicum* populations.

#### Diagnosis and relationships.

*Xiphinema penevi* sp. n. is characterised by specific combination of traits: slender body of medium size (1.54–1.85 mm), lip region rounded laterally, flattened anteriorly, separated from the body by a constriction, odontostyle 72–79 μm long, post-equatorial vulva position (V=56–58%), symbiont bacteria present in ovaria, female tail 26–32 μm long (c=50.8–61.2 and c’=1.7–1.9), conoid dorsally convex ventrally slightly concave with pointed tip, and specific ribosomal sequences (18S and ITS2). The alpha-numeric codes based on average values (ranges given in parentheses) using the polytomous key by Lamberti et al. (2004) are: A2, B3, C3 (4), D1 (2), E2, F2 (1), G2, H1, I2 (1). Subsequently described species *Xiphinema parasimile*, *Xiphinema parabrevicolle*, *Xiphinema parapachydermum*, *Xiphinema paratenuicutis* (Barsi and Lamberti 2004, Gutiérrez-Gutiérrez et al. 2012) *Xiphinema plesiopachtaicum*, *Xiphinema vallense* and *Xiphinema astaregiense* (Archidona- Yuste et al. 2016) and *Xiphinema browni* sp. n. have been also compared. Species having most similar morphometrics with *Xiphinema penevi* sp. n. were: *Xiphinema pachtaicum*, *Xiphinema plesiopachtaicum*, *Xiphinema browni* sp. n., *Xiphinema vallense* and *Xiphinema parasimile*. Due to the close relationships based on phylogenetic analyses *Xiphinema incertum*, *Xiphinema pachydermum* Sturhan, 1983 and *Xiphinema parapachydermum* were also compared. *Xiphinema penevi* sp. n. can be differentiated morphologically from:

*Xiphinema pachtaicum* by its shorter odontostyle av. 77 (72–79) *vs* 83 μm in holotype, av. 84 (78–88.5) in the present study, 89 (85–97) in females from Ethiopia, and distance of oral aperture to guide ring (68 (66–71) *vs* 78 in holotype, 77 (73–80) in the present study; shorter pharyngeal bulb (65–72 *vs* 75–80 μm) in the present study; different tail shape (conoid with gradually pointed tip *vs* conoid, subdigitate), outer cuticular layer not reaching *vs* reaching tail tip. (Lamberti and Siddiqi 1977, Getaneh et al. 2015);

*Xiphinema plesiopachtaicum* by the position of the amphideal fovea aperture (posterior *vs* at constriction level); its somewhat shorter odontostyle (72–79 *vs* 77–89 μm) and uteri (104 *vs* 138 μm); different position of the dorsal nucleus (DN in front of or at the level of DO (beginning of cuticular lining of the bulb) *vs* DN below the level of DO); different tail shape (ventrally slightly concave *vs* straight), smaller values for **c** and larger for **c**’ ratios (c=50.8–61.5 *vs* c=62.5–88.7; c’=1.7–1.9 *vs* c’=1.3–1.7);

*Xiphinema vallense* by the position of amphideal fovea (posterior constriction *vs* on the lips); its shorter body (L=1.69 (1.5–1.85) *vs* 2.01 (1.83–2.22), different position of dorsal nucleus (DN in front or at the level of DO *vs* DN below the level of DO); different tail shape (ventrally slightly concave *vs* straight) smaller values for **c** and larger values for **c**’ ratios (c=50.8–61.5 *vs* c=58.2–86.3; c’=1.7–1.9 *vs* c’=1.4–1.7), longer hyaline part (8–10 μm *vs* 6.5–8.5 μm);

*Xiphinema browni* sp. n. by its somewhat shorter body (L=1.69 (1.5–1.85) *vs* 2.03 (1.8–2.40) mm and longer bulbus (65–72 *vs* 53–69) μm; lower (2.5–4 *vs* 4–7 μm) and differently shaped lip region (not expanded *vs* expanded); different location of the dorsal nucleus (DN=9.9–12.9 % *vs* DN=12.7–21.1%); different vagina shape (funnel- *vs* bell-like Figs 16, 18);

*Xiphinema parasimile* by its somewhat shorter body (L=1.69 (1.5–1.85) *vs* 1.99 (1.75–2.26) mm in type population and avs. 1.78 -1.82 (1.56–2.04) in females from Bulgaria), different lip region shape (laterally rounded *vs* not rounded), the different location of dorsal nucleus (DN 9.9–12.9 % *vs* 13.6–18.6 %), longer bulbus (65–72 *vs* 55.5–63 μm) (Table 4); different vagina shape (funnel *vs* bell-like), structure of uteri (ovejector not present *vs* ovejector and separate uteri present) and length of uterus (36–68 vs 27–46 μm in type population and 27–39 μm in population from Bulgaria (Table 5); shorter tail (av. 29 (26–32) *vs* 33 (30.3–37.1) in the type population and 30–32 (27–35) in females from Bulgaria, c’=1.8 (1.6–1.9) *vs* 2.02 (1.79–2.28) in the type population and 2.0 (1.7–2.3) in females from Bulgaria) (Barsi and Lamberti 2004, Lazarova et al. 2008);

*Xiphinema incertum* by its different tail shape (elongate conoid *vs* bluntly conoid, ventrally slightly concave *vs* straight) and larger **c**’ values (c’=1.8 (1.6–1.9) *vs* c’=1.5 (1.4–1.7) in type material and 1.2 (0.9–1.3) in specimens from Spain, larger **a** values (a=61 (57–2-65) *vs* a=57 (56–58) in type population and a=49.7 (44.6–52.5) in the population from Spain and different vagina shape compared with females from Spain, this character not described for the type population (Lamberti et al. 1983, Gutiérres-Gutiérres et al. 2012);

*Xiphinema pachydermum* by its shorter body (L=1.69 (1.5–1.85) mm *vs* 2.24 (2.08–2.44) mm), different location of dorsal nucleus (DN=10–13 % *vs* DN=15–20%), presence of symbiont bacteria in ovaria *vs* not present; males occurrence (not present *vs* abundant);

*Xiphinema parapachydermum* by its different tail tip (not so acute and not with dorso-ventral depression) and in having symbionts in its ovaries *vs* absent, males occurrence (not present *vs* abundant).

#### Etymology.

The new species is named after Dr Lyubomir Penev, an internationally recognised publisher and authority in entomology and ecology as acknowledgement of his invaluable help and support provided to one of the authors (VP) in her research activities.

### Xiphinema pachtaicum

Taxon classificationAnimaliaDorylaimidaLongidoridae

(Tulaganov, 1938) Kirjanova, 1951

Figures 15 , 16 , 17 , 18

#### Measurements.

Tables 3–6.

#### Note.

*Xiphinema pachtaicum* has been recorded from Bulgaria and data on its morphology are available in previous studies (Lamberti et al. 1983; Peneva and Choleva 1992); here we present additional morhometric data only for the population from Balgarene together with illustrations, LM micrographs and sequence data (Table 2). It is common and associated with a wide spectrum of cultivated and wild plants (Lamberti and Siddiqi 1977).

### Xiphinema parasimile

Taxon classificationAnimaliaDorylaimidaLongidoridae

Barsi & Lamberti, 2004

Figures 15 , 17 , 18

Morphometric data and detailed description of *Xiphinema parasimile* from Bulgaria are reported previously (Lazarova et al. 2008). For the Vinogradets population two ribosomal and one mitochondrial DNA sequences were obtained (Table 2). *Xiphinema parasimile* has a limited distribution in Bulgaria (Lazarova et al. 2008).

### Sequence and phylogenetic analyses

Three rDNA sequences were obtained for the Bulgarian *Xiphinema pachtaicum* population (18S, D2-D3 and ITS2) with BLAST showing identity or very high similarity to other *Xiphinema pachtaicum* populations available at NCBI (100% for 18S, 99/100% for D2-D3 and 98% for ITS2). Further, the DNA sequences of *Xiphinema parasimile* from Vinogradets (18S, D2-D3 and *cox*1) showed highest similarity to *Xiphinema simile* from Serbia (99% for 18S), various other populations of *Xiphinema simile* and *Xiphinema opisthohysterum* (88%, D2-D3) and 78% two *cox*1 sequences – *Xiphinema pachtaicum* from the Czech Republic (GU222424) and *Xiphinema simile* from Slovakia (AM086708). The first one is the previously published sequence of *Xiphinema browni* sp. n. identified as *Xiphinema pachtaicum* (Kumari et al. 2010b). The D2 28S rDNA region was further compared to the Serbian population of *Xiphinema parasimile* (D2 part of sequences AM490214, AM490217, Barsi and De Luca 2008) and the alignment showing the different nucleotides is presented (Fig. 24). The p-distance calculated for D2 part only was 1.8–2.1% that might indicate that *Xiphinema parasimile* population from Bulgaria could represent a cryptic species.

Based on the phylogenetic analyses performed (Figs 11–15) both new species described are members of two well-supported species complexes – *Xiphinema simile* and *Xiphinema pachtaicum*. The first subgroup includes *Xiphinema simile*, *Xiphinema parasimileXiphinema browni* sp. n. and probably *Xiphinema vallense*. All occur in Europe and *Xiphinema simile* has also been reported from Central Africa (Liškova and Brown 1996, Coomans and Heyns 1997, Barsi and Lamberti 2004, Kumari 2006, Repasi et al. 2008, Lazarova et al. 2008, Bontă et al. 2012). Whether some of these records represent *Xiphinema simile* or closely related species requires new investigations using morphological discrimination and molecular markers. So far, *Xiphinema parasimile* has been recorded from the Balkan region (Barsi and Lamberti 2004, Lazarova et al. 2008, Bontă et al. 2012). *Xiphinema browni* sp. n. (previously reported as *Xiphinema pachtaicum*) seems to occur in central European countries. The second group of closely related species consists of *Xiphinema pachtaicum*, *Xiphinema penevi* sp. n., *Xiphinema incertum*, *Xiphinema parapachydermum*, *Xiphinema plesiopachtaicum*, *Xiphinema astaregiense* and *Xiphinema pachydermum*. Again, one of these species (*Xiphinema pachtaicum*) has a much wider distribution in Europe, Asia and Africa (Lamberti and Siddiqi 1977, Fadaei et al. 2003, Getaneh et al. 2015). *Xiphinema incertum* has been reported from Bulgaria, Serbia, Croaita and Spain, all other species have limited distributions – *Xiphinema plesiopachtaicum*, *Xiphinema pachydermum*, *Xiphinema parapachydermum*, *Xiphinema astaregiense*, reported only from Spain, the latter three species being amphimictic, and *Xiphinema penevi* sp. n. so far found only in north-western Africa (Sturhan 1983, Lamberti et al. 1983, Barsi and Lamberti 2002, Gutiérrez-Gutiérrez, 2012).

Based on a hierarchical cluster analysis of morphometrics Lamberti and Ciancio (1993) distinguished five species subgroups, among them the *Xiphinema pachtaicum*-subgroup (IV) consisted of 8 species with five being described from Europe (*Xiphinema fortuitum* Roca, Lamberti & Agostinelli, 1987, *Xiphinema incertum*, *Xiphinema madeirense*, *Xiphinema pachydermum* and *Xiphinema simile*), one from North America (*Xiphinema utahense* Lamberti & Bleve-Zacheo, 1979), and one from Asia (*Xiphinema opisthohysterum*). Our analyses using ribosomal and mitochondrial DNA sequences currently available in GenBank and the two new species described in this study supports the delimitation of the “*Xiphinema pachtaicum*-subgroup”, however it also includes *Xiphinema incertum*, *Xiphinema pachtaicum*, *Xiphinema pachydermum* and the recently described species *Xiphinema parapachydermum*, *Xiphinema astaregiense*, *Xiphinema plesiopachtaicum* and *Xiphinema penevi* sp. n. Phylogenetic reconstructions showed that *Xiphinema madeirense*, *Xiphinema opisthohysterum*, *Xiphinema simile* and *Xiphinema uthahense* are not part of this group, for *Xiphinema fortutium* no sequences are available. These results are in line with the findings of other recent studies on the *Xiphinema americanum*-group (Gutiérrez-Gutiérrez et al. 2012, Archidona-Yuste et al. 2016). *Xiphinema simile* (presented by two types of sequences for populations from Serbia and the Czech Republic in 18S rDNA and *cox*1 trees), *Xiphinema parasimile* and *Xiphinema browni* sp. n. formed a separate subgroup outside the *Xiphinema pachtaicum*-subgroup, so far consisting only of parthenogenetic species. Therefore we proposed this clade to be referred as the *Xiphinema simile*-subgroup. The recently described species *Xiphinema vallense* seems also evolutionary very closely related to this subgroup because of its high morphometric and DNA similarity, however amplifying additional sequences for other molecular markers (e.g. 18S and *cox*1) could help to clarify its relationships.

## Supplementary Material

XML Treatment for Xiphinema browni

XML Treatment for Xiphinema penevi

XML Treatment for Xiphinema pachtaicum

XML Treatment for Xiphinema parasimile
